# Direct Formation of Karst Fenglin-like Cu-Based Metal–Organic Framework on CuSCN Hole Transport Layer for Highly Efficient and Stable Perovskite Solar Cells

**DOI:** 10.1021/acsami.6c06348

**Published:** 2026-05-12

**Authors:** Nideesh Perumbalathodi, Sarin Sajjadu Krishna, Tzu-Sen Su, Tzu-Chien Wei

**Affiliations:** † Department of Chemical Engineering, 34881 National Tsing Hua University, 101, Section 2, Kuang Fu Road, Hsinchu 30013, Taiwan (R.O.C.); ‡ Graduate Institute of Energy and Sustainability Tech, 34878 National Taiwan University of Science and Technology, No.43, Keelung Rd., Sec. 4, Da’an Dist., Taipei City 106335, Taiwan (R.O.C.); § Research Center for Critical Issues, Academia Sinica, South Campus, No 100, Section 1, Guiren 13th Road, Guiren District, Tainan City 711010, Taiwan (R.O.C.)

**Keywords:** CuSCN inorganic HTM, conductive-metal−organic framework for PSC, hybrid inorganic HTM, in situ MOF films, interfacial engineering, perovskite solar cell

## Abstract

Improving charge extraction efficiency and minimizing interfacial recombination losses are critical to enhancing the performance of inverted perovskite solar cells (PSCs). Although copper thiocyanate (CuSCN) exhibits excellent hole transport capability, its interfacial reactivity and poor contact with the perovskite (PVSK) layer result in inefficient hole extraction and severe interfacial recombination, significantly limiting device efficiency and stability. In this study, we present a novel strategy to directly grow a three-dimensional metal–organic framework (MOF), Cu_3_(HHTP)_2_, in situ on wet-coated CuSCN films. This approach enables the construction of a hybrid CuSCN/Cu_3_(HHTP)_2_ hole transport layer that significantly improves interfacial quality and enhances charge transport characteristics. Experimental results demonstrate that PSCs incorporating this hybrid HTM structure achieve a maximum power conversion efficiency (PCE) of 20.05%, substantially outperforming unmodified CuSCN-based devices (16.10%) and exhibiting superior operational stability.

## Graphical abstract

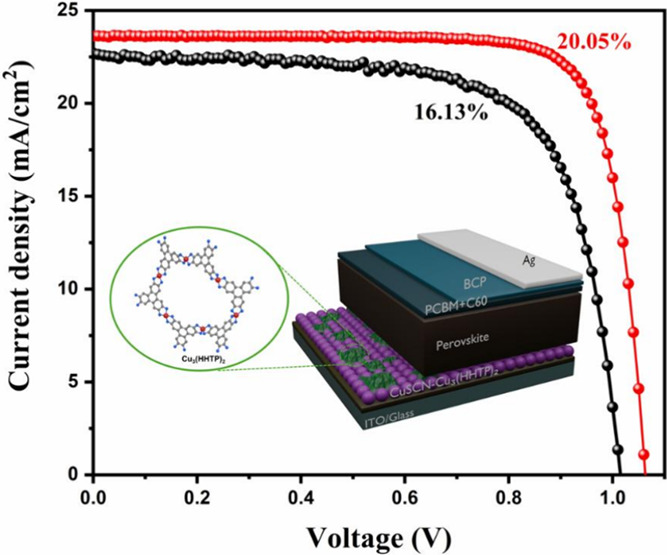

## 1 Introduction

The hybrid organic–inorganic lead halide perovskite (PVSK) materials have emerged as a strong contender for next-generation photovoltaic technologies due to their exceptional properties, including long carrier diffusion lengths, high absorption coefficients, and high defect tolerance.^1–6^ The device configuration of perovskite solar cells (PSCs) is generally categorized into the conventional n–i–p architecture and the inverted p–i–n architecture. The p–i–n PSC offers distinct advantages over the n–i–p structure, such as improved process flexibility, facile integration with tandem solar cells,^7–11^ and enhanced stability under operational conditions.^12–14^ Over the past decade, through the concerted efforts of researchers, inverted PSCs have achieved power conversion efficiencies (PCE) of over 26%, highlighting their potential to stand out among the various configurations of PSCs.^15–17^

A key factor influencing the performance of inverted PSCs is the optimization of the hole transport material (HTM) that is initially deposited on the transparent conductive substrate. Due to its excellent optical transparency, high thermal stability, low cost, and suitable energy band alignment with the mainstream PVSK absorber layer, copper thiocyanate (CuSCN) has been studied as a promising HTM for n–i–p PSCs.^18–21^ However, despite the numerous advantages, CuSCN-based HTMs suffer from several interfacial issues between the PVSK and CuSCN. These drawbacks hinder its widespread application in inverted PSCs and lead to the subpar photovoltaic performance of the device, compromising its competitiveness with other materials such as NiO*x*,^17,22–24^ self-assembled monolayers (SAMs)^25–27^ and so on.

Initial attempts to utilize CuSCN in inverted PSCs encountered many setbacks and challenges. Subbiah et al., electrodeposited CuSCN as the HTM in inverted PSCs, but this resulted in only 3.8% PCE due to excessively high series resistance from the thick CuSCN layer.^28^ Subsequently, Zhao et al., utilized spin coating followed by low-temperature drying (60 °C) to prepare a more uniform CuSCN layer, which reduced the series resistance and improved device performance.^29^ Based on this, Ye et al., adopted a one-step rapid deposition crystallization method to prepare the PVSK layer, achieving a PCE of 16.6%. This method effectively reduced the surface roughness of CuSCN film and interfacial contact resistance, leading to better charge extraction and reduced recombination losses.^30^ Furthermore, the dual-layer HTM configuration combining organic materials with CuSCN has also been explored. Xiong et al., reported an inverted PSC using a bilayer HTM composed of CuSCN-modified PEDOT:PSS film. The modification of CuSCN enhanced the energy band alignment between the PVSK and PEDOT: PSS, resulting in a device PCE of 10.09%. In comparison, the control device using single-layer PEDOT:PSS achieved a PCE of 9.1%. This modification improved both the charge extraction efficiency and the overall device stability.^31^ Inspired by these results, researchers began to actively explore the strategy of composite HTMs to overcome the limitations of single-layered CuSCN HTM. Wang et al., demonstrated that a CuSCN/CuI composite HTM could significantly enhance photovoltaic performance, achieving a PCE of 18.76%. The composite structure benefited from the complementary properties of CuSCN and CuI, with CuSCN playing a crucial role in forming high-quality HTM thin films that improve device efficiency by reducing interfacial defects and enhancing charge transport.^32^

In addition, the wet processability of CuSCN film has been criticized for a long time. Wet-processed CuSCN HTMs typically require highly polar organic solvents such as diethyl sulfide (DES) or dipropyl sulfide (DPS). These solvents not only have an unpleasant odor but are also environmentally unfriendly, posing challenges to the scalability and sustainable production of CuSCN-based PSCs. To address this issue, Wijeyasinghe et al., developed a water-based processing technique for CuSCN HTM, achieving a PCE of 17.5%. They utilized eco-friendly water-based precursors and low-temperature processing at room temperature, offering a more sustainable alternative for the fabrication of CuSCN.^33^ A recent breakthrough was achieved by Liang et al., who enhanced the performance of CuSCN films by a chlorination treatment using a dry etching system under controlled Cl_2_ gas exposure. The results indicated that Cl_2_ doping improved the conductivity of the CuSCN films, boosting the PCE of inverted PSCs using CuSCN as HTM to over 20%. Furthermore, these Cl_2_-doped CuSCN HTMs exhibited stronger resistance to degradation under illumination and high temperatures compared to undoped CuSCN and the control PEDOT:PSS HTM.^34^ From these studies, it is evident that two critical challenges must be addressed for CuSCN to function as a viable HTM in scalable PSC technologies: (i) the development of environmentally benign, alkyl sulfide-free film-processing routes and (ii) effective mitigation of interfacial defects at the CuSCN/PVSK interface. The solvent-related limitations associated with DES are addressed in this work by replacing DES with a process-friendly aqueous ammonia-based aqueous solvent.^33,35^ As discussed earlier, the intrinsic chemical reactivity of the CuSCN/PVSK interface has motivated the insertion of insulating polymer interlayers to suppress interfacial degradation. However, while these insulating layers can chemically passivate the interface, they inevitably compromise interfacial charge transport, leading to charge accumulation and degraded device performance.^36–38^ These limitations highlight the urgent need for conductive and chemically robust interfacial engineering strategies that stabilize the CuSCN/PVSK interface without sacrificing hole-transport properties.

In this context, our search for an electrically benign yet chemically stable interfacial modifier led us to an emerging class of materials known as metal–organic frameworks (MOFs). MOFs are crystalline porous frameworks constructed from metal ions coordinated with organic linkers and are distinguished by their high chemical tunability, structural diversity, and thermal stability. To date, several studies have explored the integration of presynthesized MOFs at either the ETM­(electron transporting material)/PVSK or PVSK/HTM interfaces. For example, Shen et al., introduced a zeolitic imidazolate framework-8 (ZIF-8) interlayer on mesoporous TiO_2_ ETM, where the MOF scaffold regulated PVSK crystallization and promoted grain growth.^39^ Similarly, Liu et al., employed a Pb-based postmetalated MOF-525 between compact TiO_2_ and PVSK, providing heterogeneous nucleation sites that improved crystal quality and reduced grain-boundary defects.^40^ At the PVSK/HTM interface, Lee et al., incorporated UiO-66 and MOF-808 at the NiO_*x*_/PVSK junction, effectively modulating PVSK crystallization kinetics, enlarging grain size, reducing trap density, and improving the PCE from 15.79 to 17.01%.^41^

Despite these promising results, MOF integration in PSCs has predominantly relied on ex situ synthesis and dispersion of preformed MOF particles, which introduces several intrinsic drawbacks. Precise control over MOF particle size and dispersibility remains challenging, often leading to aggregation, rough interfaces, and nonuniform film coverage that are detrimental to charge transport and device reproducibility. In addition, coating methods for MOF-based interlayers are still immature, frequently resulting in incomplete coverage and additional recombination pathways. The intrinsically low electrical conductivity of many MOFs further increases series resistance and limits the fill factor (FF), while repeated MOF synthesis, purification, and redispersion complicate fabrication and hinder scalability. Collectively, these issues restrict the practical implementation of MOFs in large-area PSC manufacturing.

To overcome these limitations, we propose a novel in situ interfacial growth strategy that directly integrates a conductive MOF between the CuSCN HTM and the PVSK absorber. Specifically, we employ the two-dimensional conductive MOF tricopper­(II) bis­(2,3,6,7,10,11-hexahydroxytriphenylene), Cu_3_(HHTP)_2_, which forms spontaneously at the CuSCN surface via a bottom-up interfacial reaction between Cu^2+^ species released from CuSCN and the HHTP ligand in solution. This process yields a conformal Cu_3_(HHTP)_2_ layer that is intimately coupled with the CuSCN substrate, forming a CuSCN/Cu_3_(HHTP)_2_ hybrid HTM.

This in situ interfacial formation offers several decisive advantages over conventional MOF-integration strategies. It eliminates the need for presynthesized MOF particles, thereby avoiding aggregation and dispersion issues, and ensures seamless interfacial contact without introducing additional defects. The intrinsically conductive nature of Cu_3_(HHTP)_2_ facilitates efficient hole extraction and favorable energy-level alignment with the PVSK absorber, minimizing resistive losses. Moreover, the simple solution-based growth process enhances reproducibility and scalability, key prerequisites for industrial translation. In effect, this strategy provides a promising pathway toward high-efficiency, stable, and environmentally sustainable inverted PSCs based on CuSCN HTMs.

## 2 Experimental Section

### 2.1 Deposition of CuSCN Film

ITO glass (15 Ω/sq, Lumtec, Taiwan) was cleaned by sonicating in a diluted detergent solution, followed by rinsing with DI water, acetone, and isopropanol, sequentially for 30 min in each. Then the substrates were subjected to UV-Ozone treatment for 30 min to enhance the surface wettability and remove organic contaminants. A CuSCN solution was prepared by dissolving commercial CuSCN powder (≥99%, Sigma-Aldrich) in a 50 vol % aqueous NH_3_ (Alfa Aesar, ≥98%) solution at a concentration of 15 mg/mL. The solution was stirred at 40–50 °C for 2 h to ensure complete dissolution. Undissolved CuSCN particles were then removed by filtration through a 0.22 μm nylon syringe filter. The prepared CuSCN solution was then spin-coated onto the cleaned ITO glass at a spin rate of 3000 rpm for 25 s in a dry room (temperature and humidity of 15 ± 5% and 25 ± 1 °C). Subsequently, the deposited CuSCN films were dried at 100 °C for 15 min.

### 2.2 Formation of Cu_3_(HHTP)_2_ MOF on CuSCN Film

As-deposited CuSCN films were immersed in a 0.5 mM HHTP ethanolic solution (>95.0%, TCI Japan) for durations ranging from 0.5 to 4 min to form a Cu_3_(HHTP)_2_ MOF layer on the CuSCN surface. Following the completion of immersion, the samples were rinsed with absolute ethanol to remove any residual HHTP molecules from the surface. Finally, the samples were mildly dried at 40 °C for 5 min to remove any residual solvent within the newly formed MOF structures.

Notably, while previous reports have primarily utilized conventional copper salts as the copper precursor in the synthesis of Cu_3_(HHTP)_2_ MOF, no studies have investigated the use of CuSCN in this context. Therefore, in this work, we have also explored the interaction between CuSCN and HHTP. Cu_3_(HHTP)_2_ powder was synthesized by the reaction between CuSCN solution and HHTP powder. (hereafter referred to as Cu_3_(HHTP)_2_ powder). For comparison, a reference Cu_3_(HTTP)_2_ powder made from CuSO_4_ precursor was synthesized.^42^ (hereafter referred to as ref-Cu_3_(HHTP)_2_ powder) Specifically, 0.5 mM CuSO_4_.5H_2_O and 0.3 mM HHTP powder were dispersed in 10 mL DI water in a beaker. After ultrasonication for 5 min, 4 μL of ammonia was added to the solution, which was then stirred for 5 h to form the Cu_3_(HHTP)_2_ powder. The powders were then collected, washed with DI water, ethanol and acetone, and dried at 70 °C for 12 h.

### 2.3 PSC Fabrication

PVSK precursor solution with a stoichiometry of Cs_0.05_((FA_0.84_MA_0.16_))_0.95_Pb­(I_0.84_Br_0.16_)_3_ was prepared by dissolving 522.45 mg of PbI_2_, 79.2 mg of PbBr_2_, and 17.44 mg of CsI in 1 mL of a DMF/DMSO solvent mixture (8:2 volume ratio). The solution was stirred and heated at 70 °C for 10 min. Subsequently, 185.26 mg of formamidine iodide (FAI) and 22.97 mg of methylammonium bromide (MABr) were added to the solution, followed by stirring for 3 h within a glovebox. The prepared PVSK solution was then spin-coated onto the HTM-coated substrates using a two-step process. The first step involved a spin rate of 2000 rpm for 2 s, followed by a ramp-up step to 4500 rpm for 35 s. During the final 16 s of the spin coating program, 250 μL of anhydrous ethyl acetate was dripped onto the spinning film to induce rapid crystallization and improve film morphology. The spin-coated PVSK film was then annealed at 115 °C for 10 min to complete the PVSK crystallization process. Following PVSK deposition, the electron transporting material (ETM) was fabricated by spin-coating a solution of 25 mg of Phenyl-C_61_-butyric acid methyl ester (PCBM) and 5 mg of C_60_ in 1 mL of CB at 2000 rpm for 30 s. Subsequently, a hole-blocking layer of 0.5 mg of bathocuproine (BCP) in anhydrous IPA at 4500 rpm for 20 s. Finally, a 100 nm-thick Ag electrode was thermally evaporated onto the device.

### 2.4 Characterizations

The topography of the thin films was examined using field-emission scanning electron microscopy (FESEM, SU8010, Hitachi, Japan). Cross-sectional analysis of the CuSCN and CuSCN-MOF layers was performed using focused ion beam (FIB) microscopy (FEI Helios Nanolab 600i). The surface roughness of the films was measured using atomic force microscopy (AFM, C3000, Nanosurf, Switzerland). The valence band (VB) position was determined using ultraviolet photoelectron spectroscopy (UPS, PHI 5000 Versa Probe II, USA) with helium (He) as the excitation source at an energy of 21.2 eV. The optical bandgap of the thin films was calculated from ultraviolet–visible (UV–vis) absorption spectra obtained using a UV–vis spectrometer (USB2000, Ocean Optics, USA). Crystallographic analysis was performed using X-ray diffraction (XRD) with a Rigaku Ultima IV diffractometer equipped with a ceramic tube using copper K-alpha (Cu Kα) radiation (λ = 1.5418 Å). Out-of-plane crystallographic analysis of the thin film was conducted using grazing incidence X-ray diffraction (GIXRD) with a Bruker D8 advance diffractometer equipped with a Cu Kα radiation source (λ = 1.5406 Å).

Photoluminescence properties, including steady-state photoluminescence (SSPL) and time-resolved photoluminescence (TRPL), were investigated using a photoluminescence spectrometer (FL900, EDINBURGH Instruments, England). Surface contact potential difference (CPD) was monitored using Kelvin probe microscopy­(KPFM,C3000, Nanosurf, Switzerland). Hole mobility (μ) and the trap-filled limit voltage (*V*_TFL_) of hole-only devices were determined using space-charge-limited current (SCLC) measurements performed with a source meter (Keithley 2400, USA) at a scan rate of 20 mV/s over a voltage range of 0–5 V. The photovoltaic performance of PSCs was evaluated using a computer-controlled digital source meter (Keithley 2400, USA) in conjunction with a commercial solar simulator (PEC-L15, Peccell Technologies, Japan) under standard test conditions of 1 Sun illumination (AM1.5G). Before recording the current–voltage (*J*–*V*) curve, the light intensity was calibrated using a monocrystalline silicon photodiode (KG3, Oriel, USA), and the illuminated area was precisely controlled using a 0.1 cm^2^ photo mask. The external quantum efficiency (EQE) spectra were measured using a PEC-S20 system (Peccell Technologies, Japan). Electrochemical impedance (EIS) measurements for the PSC devices were performed in the dark using an Auto lab PGSTAT302N workstation, sweeping frequencies from 0.1 Hz to 100 kHz.

## 3 Results and Discussion

### 3.1 Synthesis and Characterization of Cu_3_(HHTP)_2_ MOF from CuSCN

We first studied the chemical interactions between CuSCN and the HHTP ligand. Figure S1 presents the Raman spectra of CuSCN powder, Cu_3_(HHTP)_2_ powder, and reference Cu_3_(HHTP)_2_ powder. The CuSCN spectrum exhibits characteristic peaks at 255, 432, 746, and 2172 cm^–1^, corresponding to the vibrational modes of Cu–N, S–C≡N, C–S bonds, and Cu–SCN, respectively.^43^ Notably, the Raman spectra of both the Cu_3_(HHTP)_2_ and reference Cu_3_(HHTP)_2_ powders also show these peaks, in addition to prominent bands at 1366 and 1566 cm^–1^, which are assigned to the D and G bands of the MOF. These additional peaks indicate the presence of sp^2^-hybridized carbon, characteristic of the 2D Cu_3_(HHTP)_2_ framework.^44^ These observations strongly support that the introduction of the HHTP ligand to CuSCN successfully leads to the formation of the Cu_3_(HHTP)_2_ MOF.

The Cu_3_(HHTP)_2_ MOF formation, in this case, is believed to be initiated by Cu^2+^ ions, formed as a result of CuSCN dissolution in NH_3_ solution, which likely takes place through a pale blue Cu^2+^-amine complexation, as evidenced by the color change. (Figure S2). Due to the electron-deficient nature of Cu^2+^ ions, it can readily coordinate with the electron-rich HHTP ligand. This coordination between the Cu^2+^ ions and HHTP ligands ultimately leads to the formation of the Cu_3_(HHTP)_2_ MOF structure, as represented by the following equation:^44^

$$3{\mathrm{Cu}}^{2+}+2{\mathrm{HHTP}}^{-3}=Cu_{3}{\left(\mathrm{HHTP}\right)}_{2}$$

Upon successfully demonstrating CuSCN/MOF formation in bulk solution, the investigation was extended to CuSCN thin films. To elucidate the mechanism of in situ MOF growth at the CuSCN interface, CuSCN films spin-coated onto ITO substrates were immersed in 20 mL of ethanol for 0.5, 1, 2, and 4 min, denoted as CuSCN-0.5M, CuSCN-1M, CuSCN-2M, and CuSCN-4M, respectively. The corresponding ethanol solutions were subjected to UV–Vis analysis to track Cu^2+^ leaching from the films (Figure S3a). Even after just 0.5 min of ethanol immersion, a characteristic Cu^2+^ absorption band appeared at ∼210 nm, and its intensity progressively increased with immersion duration. This behavior confirms the gradual release of Cu^2+^ ions from the CuSCN film into the ethanolic medium, implying the formation of Cu^2+^ nucleation sites capable of participating in subsequent coordination reactions.

To validate this hypothesis, the experiment was repeated using a 0.5 mM HHTP ethanolic solution under identical conditions. The resulting UV–Vis spectra (Figure S3b) exhibited two distinct features at ∼360 and ∼660 nm, corresponding to the organic ligand’s n−π* transition and the ligand-to-metal charge-transfer (LMCT) process, respectively. Both absorption bands intensified with dipping time, indicating continuous Cu-HHTP coordination and confirming MOF formation directly within the solution. The time-dependent increase in MOF-related absorption correlates strongly with the Cu^2+^-leaching behavior observed in Figure S3b, demonstrating that the extent of MOF formation scales with the amount of Cu^2+^ released from the CuSCN film.^45^

For PSC device fabrication, CuSCN-coated ITO substrates were immersed in a 0.5 mM HHTP ethanolic solution for 0.5, 1, 2, and 4 min, referred to as CuSCN-0.5MOF, CuSCN-1MOF, CuSCN-2MOF, and CuSCN-4MOF. In situ UV–Vis monitoring was performed directly on the CuSCN/ITO substrates during the dipping process (Figure 1a). Two primary absorption bands were observed: a sharp n−π* transition centered at ∼350 nm and a broad LMCT band at ∼630 nm. Both bands increased systematically as the reaction proceeded (0–270 s). The progressive enhancement of the LMCT band, arising from Cu-HHTP coordination, signifies the increasing incorporation of Cu^2+^ into the forming metal–organic framework. This time-dependent spectral evolution confirms continuous nucleation and growth of the Cu-based MOF directly on the CuSCN surface, establishing an interfacial hybrid structure critical for subsequent PSC performance enhancement.

**Figure 1:**
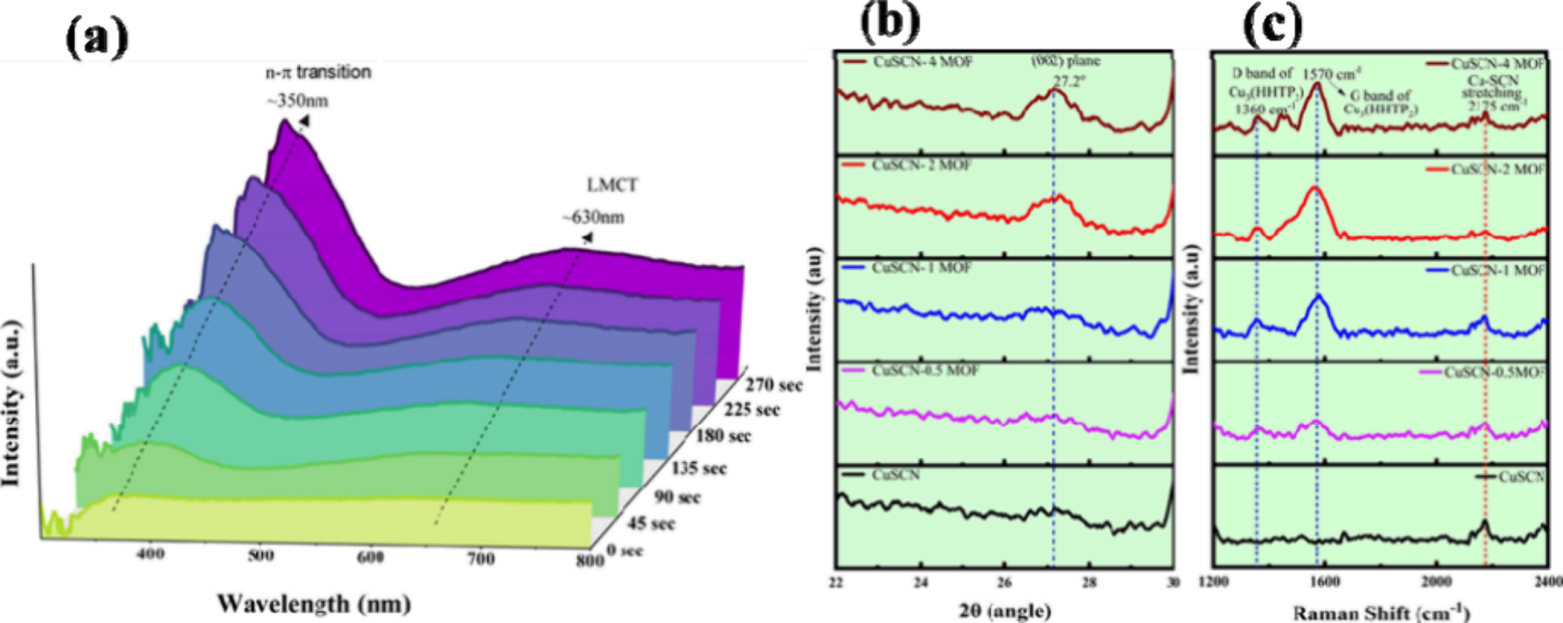
(a) In situ UV–vis spectra, (b) out-of-plane GIXRD patterns, and (c) Raman spectra of the CuSCN film immersed in an HHTP solution for varying durations.

This observation was further supported by a visible color change in the CuSCN film from transparent to pale blue (Figure S4a). It can be observed that there is a gradual decrease in transmittance with increasing immersion time (Figure S4b). Notably, a significant reduction in transmittance was observed in CuSCN-4MOF, suggesting the thickening of the MOF layer. Such a decrease in transmittance may hinder light penetration to the underlying PVSK layer in photovoltaic devices, potentially reducing device performance. To confirm the formation of Cu_3_(HHTP)_2_ on the CuSCN film surface, out-of-plane GIXRD analysis was performed (Figure 1b). The characteristic MOF peak was detected at 27.2° which corresponds to the (002) planes of Cu_3_(HHTP)_2_ MOF, indicating successful growth of crystalline MOF on the CuSCN surface.^46^ However, these characteristic peaks were absent for CuSCN-0.5MOF and CuSCN-1MOF, suggesting that short immersion time may result only in MOF seeding rather than the formation of crystalline MOF particles. The Raman spectra of these films (Figure 1c) also confirm the seeding and subsequent growth of MOF particles over the CuSCN film. Raman spectra of pristine CuSCN film exhibit its characteristic peak at 2175 cm^–1^ characteristic vibrational peak of Cu-SCN bond, with the remaining peaks of CuSCN masked by ITO peaks. As the immersion time extends, we can see the growth of crystalline MOF particles as evidenced by the appearance of sharp peaks at 1360 and 1570 cm^–1^.

XPS analysis was conducted to investigate the chemical interactions between the HHTP ligand and CuSCN films for various immersion times. The pristine CuSCN film, serving as a baseline (Figure 2a), shows well-defined carbon peaks. The C–C/H peak at 284.34 eV corresponds to adventitious carbon, while the peak at 285.10 eV is attributed to C–S bonding, and 286.10 eV represents the SCN group in the CuSCN film.^33,43,35^ For CuSCN-0.5MOF (Figure 2b), notable spectral changes were observed. The C–C/H peak shifts slightly to 284.41 eV, indicating initial interaction between the HHTP ligand and the CuSCN surface. New peaks emerge at 285.55 and 288.10 eV, corresponding to phenolic hydroxyl carbon from the HHTP ligand and metal-coordinated carbon (Cu–C–O), respectively. These features suggest early stage ligand adsorption with weak surface coordination. In CuSCN-1MOF (Figure 2c), further surface modification is evident. The C–C/H peak remains at 284.41 eV but shows noticeable broadening, implying stronger ligand–surface interactions. The phenolic OH peak intensifies at 285.88 eV, and the peak at 288.50 eV, attributed to Cu–O–C coordination bonds, becomes more prominent, confirming enhanced ligand binding to CuSCN.

**Figure 2:**
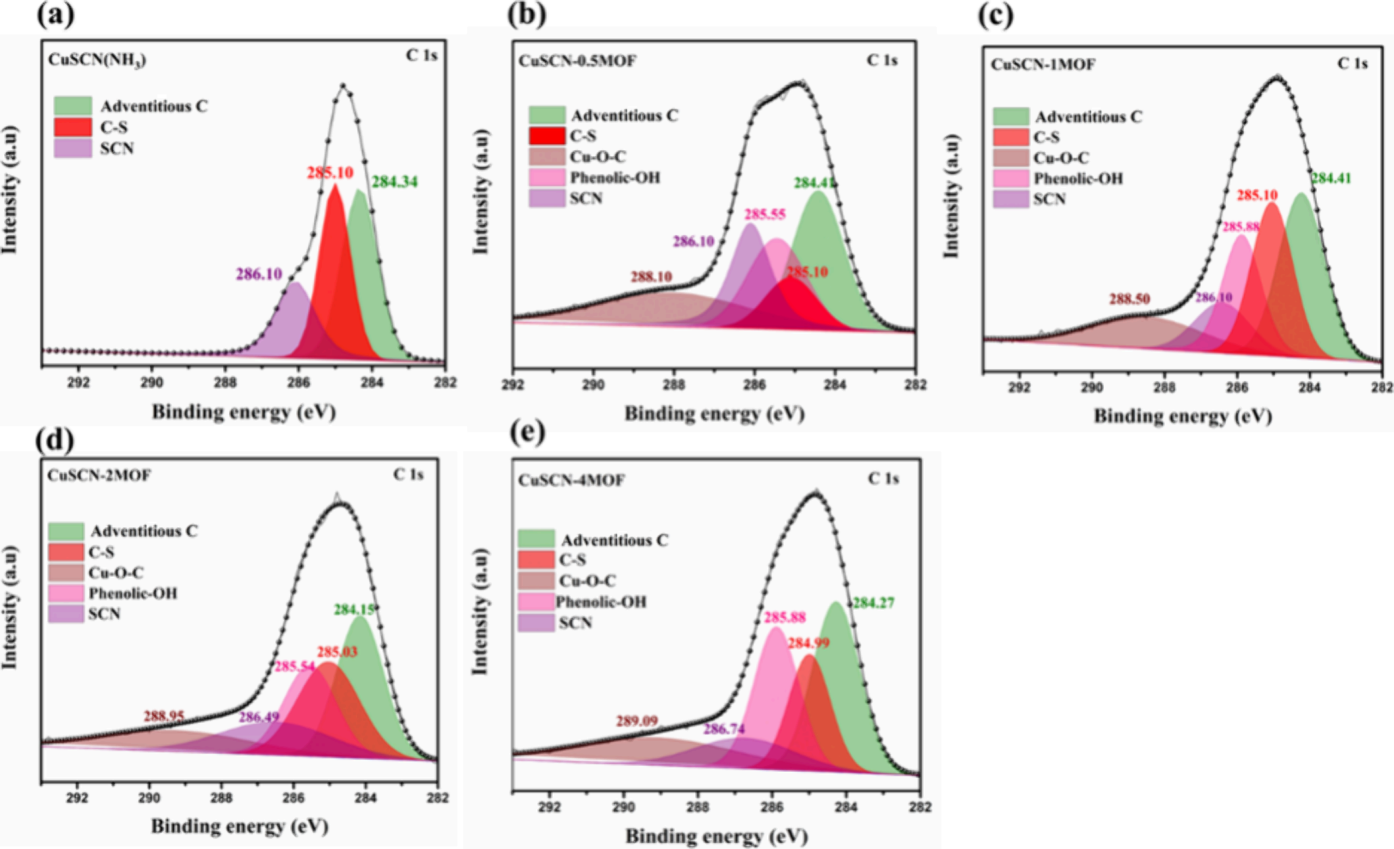
XPS C 1s spectra of (a) CuSCN, (b) CuSCN-0.5MOF, (c) CuSCN-1MOF, (d) CuSCN-2MOF, and (e) CuSCN-4MOF.

For CuSCN-2MOF (Figure 2d), the interaction becomes more pronounced. The C–C/H peak shifts further to 284.15 eV, and the peak at 285.54 eV is consistent with phenolic carbon species. The peak at 288.99 eV confirms the formation of strong Cu–O–C coordination bonds, indicative of extensive surface modification and complete ligand coverage. In CuSCN-4MOF (Figure 2e), the spectral features stabilized. The C–C/H peak remains at 284.27 eV. The persistent phenolic carbon peak at 285.88 eV and the intensified Cu–O–C peak at 289.1 eV confirm full surface coverage by the HHTP ligand. The minimal spectral differences between CuSCN-2MOF and CuSCN-4MOF suggest that surface coordination has reached equilibrium.

The Cu 2p core-level spectra of pristine CuSCN and CuSCN-0.50MOF, CuSCN-1MOF, CuSCN-2MOF and CuSCN-4MOF reveal a systematic chemical evolution at the HTM interface (Figure 3). Both the Cu 2p_3/2_ and Cu 2p_1/2_ regions show progressive B.E. shifts and variations in the Cu^+^/Cu^2+^ ratio, reflecting valence redistribution and coordination chemistry induced by ligand interaction. For pristine CuSCN, the Cu^+^ 2p_3/2_ and 2p_1/2_ peaks appear at 932.45 and 952.30 eV, respectively, consistent with reported Cu­(I)–thiocyanate environments­(Figure 3a). A substantial Cu^2+^ contribution is also detected at 934.02 eV (2p_3/2_) and 953.46 eV (2p_1/2_), accounting for ∼ 47.5% of total Cu (Table 1). The corresponding Cu^2+^ satellite peak appears at 943.49 eV, further corroborating the presence of Cu^2+^ species.

**Figure 3:**
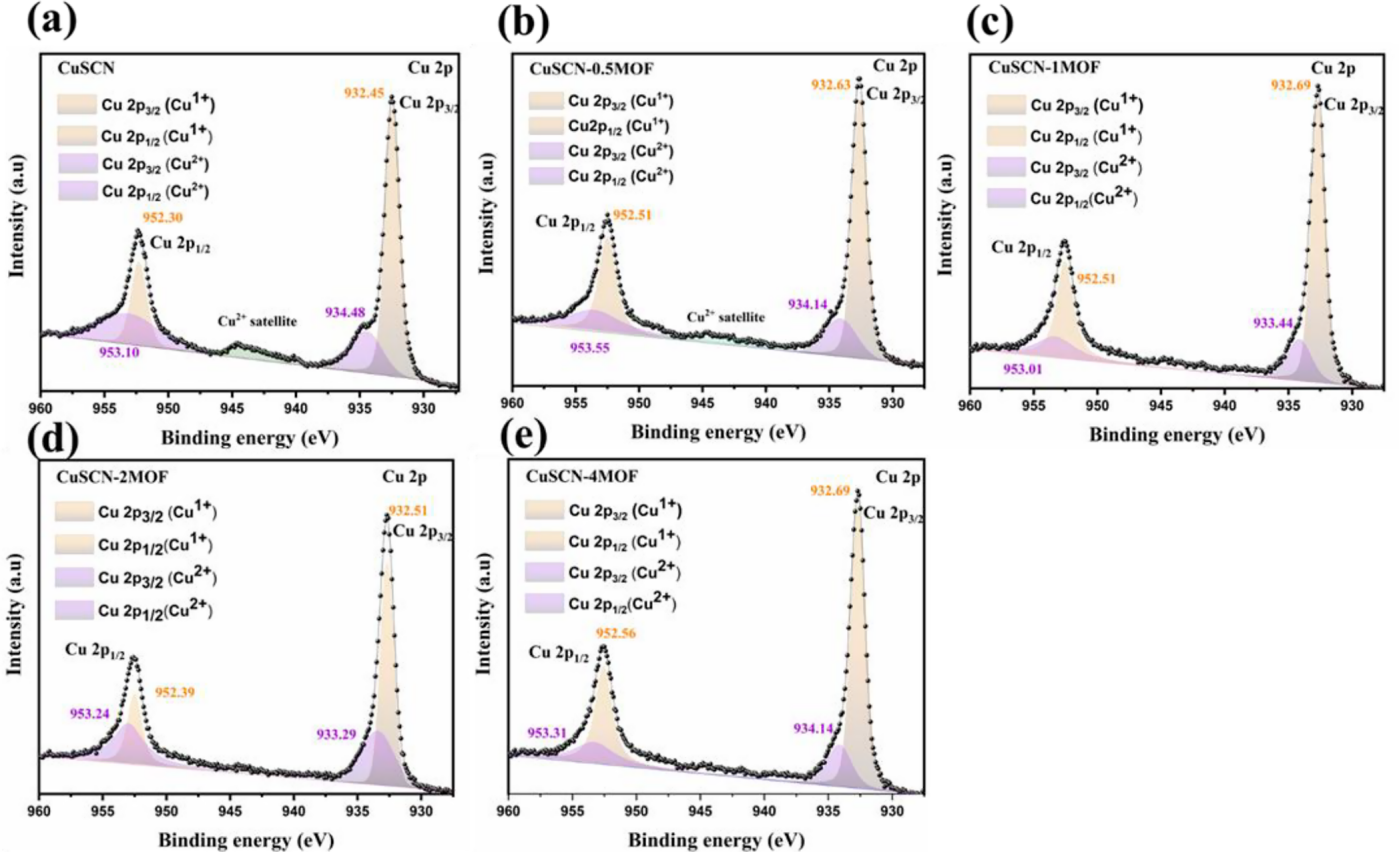
XPS Cu 2p spectra of (a) CuSCN, (b) CuSCN-0.5MOF, (c) CuSCN-1MOF, (d) CuSCN-2MOF, and (e) CuSCN-4MOF.

**Table 1:** Cu^2+^/Cu^+^ Ratio of CuSCN Films Immersed in HHTP Solution for Different Durations

sample name	Cu^+^ (2p_3/2_) B.E (eV)	Cu^+^ (2p_1/2_) B.E (eV)	Cu^+^ area	Cu^2+^(2p_3/2_) B.E (eV)	Cu^2+(^2p_1/2_) B.E (eV)	Cu^2+^ area	Cu^2+/^Cu^+^ ratio	% Cu^2+^
CuSCN	932.45	952.30	49,694	934.02	953.46	44,897	0.903	47.50%
CuSCN-0.5MOF	932.55	952.41	47,628	934.02	953.53	40,749	0.811	44.80%
CuSCN-1MOF	932.63	952.51	48,253	934.14	953.55	22,792	0.473	31.80%
CuSCN-2MOF	932.74	952.66	40,072	934.19	953.68	20,732	0.414	29.30%
CuSCN-4MOF	932.99	952.92	43,804	934.54	954.00	12,340	0.279	21.80%

This mixed-valence character arises from surface oxidation and/or partial disproportionation into [Cu­(II)–amine]^2+^ complexes, in agreement with earlier reports on CuO and Cu­(OH)_2_ formation.

For CuSCN-0.5MOF film­(Figure 3b), the Cu^+^ peaks shift slightly to 932.55 eV (2p_3/2_) and 952.41 eV (2p_1/2_), while the Cu^2+^ features remain at 934.02/953.53 eV. The Cu^2+^ satellite peak correspondingly shifts to 943.76 eV. The Cu^2+^ fraction decreases marginally to 44.8%. These results indicate the early onset of coordination between HHTP hydroxyl groups and surface copper sites. The small positive shift in binding energies suggests a more electron-withdrawing ligand environment, although the overall oxidation-state distribution remains comparable to pristine CuSCN.

A pronounced transformation is observed in the CuSCN-1MOF film­(Figure 3c). The Cu^+^ components shift to 932.63 and 952.51 eV, while the Cu^2+^ features shifted to 934.14 and 953.55 eV. Importantly, the Cu^2+^ fraction drops sharply to 31.8%, corresponding to a nearly 2-fold decrease in the Cu^2+/^Cu^+^ ratio (0.473). This reduction may be attributed to (i) electron donation from HHTP ligands, reducing Cu^2+^ to Cu^+^, or (ii) preferential coordination of Cu^2+^ oxides, leading to the formation of Cu-HHTP complexes. These mechanisms are consistent with the nucleation of a Cu–HHTP coordination layer, where Cu^+^ becomes the dominant oxidation state.

For CuSCN-2MOF film (Figure 3d), the Cu^+^ peaks further shift to 932.74 and 952.66 eV, while the Cu^2+^ features appear at 934.19 and 953.68 eV. The Cu^2+^ fraction decreases to 29.3%, confirming the trend toward Cu^+^ enrichment. The incremental shift of ∼0.3 eV relative to pristine CuSCN reflects the embedding of Cu centers in an oxygen-rich, electronegative coordination environment provided by HHTP.

After 4 min of dipping (CuSCN-4MOF, Figure 3e), the Cu^+^ peaks are observed at 932.99 and 952.92 eV, while the Cu^2+^ peaks shift to 934.54 and 954.00 eV. Both sets of features exhibit ∼+0.5 eV shifts relative to pristine CuSCN. The Cu^2+^ fraction is further reduced to 21.8%, corresponding to a Cu^2+^/Cu^+^ ratio of only 0.279. This result confirms that the surface is now overwhelmingly dominated by Cu^+^ species, with residual Cu^2+^ progressively suppressed. The stabilization of Cu^+^ can be attributed to the formation of a robust CuSCN/Cu-HHTP hybrid interface, where Cu^+^ sites are effectively protected from oxidation by ligand coordination. The progressive reduction in Cu^2+^ signal intensity and the stabilization of Cu^+^ species highlight the efficient formation of surface-bound MOF structures and the chemical transformation of the CuSCN interface.^35,47^

The O 1s XPS spectra reveal progressive ligand coordination between the HHTP solution and the CuSCN film during immersion. Initially, the pristine CuSCN film deposited on ITO glass (Figure 4a) displays peaks corresponding to free hydroxyl groups at 533.30 eV and C–OH/C–C bonds at 532.47 eV, consistent with previous reports.^33^ After 30 s of immersion (Figure 4b), a new peak appears at 530.90 eV, which may be attributed to Cu–O species formed by the partial oxidation of Cu^+^ centers. Additionally, a peak at 531.68 eV is assigned to phenolate-type Cu–O–C bonding, indicating coordination of deprotonated hydroxyl groups from HHTP to Cu^2+^, suggesting the initiation of Cu_3_(HHTP)_2_ MOF formation.^35–50^

**Figure 4:**
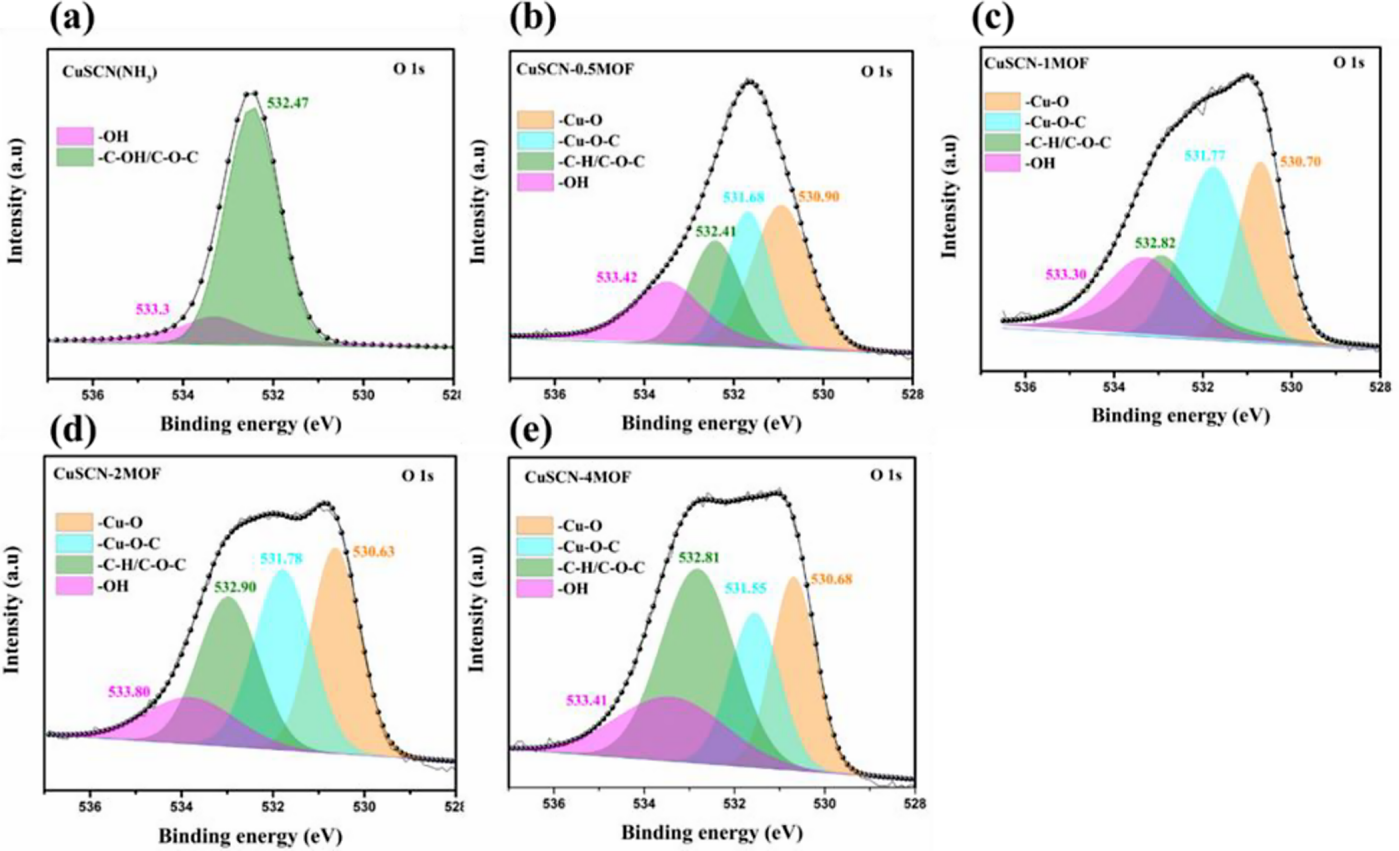
XPS O 1s spectra of (a) CuSCN, (b) CuSCN-0.5MOF, (c) CuSCN-1MOF, (d) CuSCN-2MOF, and (e) CuSCN-4MOF.

After 1 min (CuSCN-1MOF, Figure 4c) and 2 min (CuSCN-2MOF, Figure 4d) of immersion, peaks corresponding to Cu–O (530.70 and 530.63 eV) and Cu–O–C (531.77 and 531.78 eV) become more pronounced and stabilized, indicating enhanced ligand interaction and a reduction in surface defects. In CuSCN-4MOF (Figure 4e), the spectra reach a steady state, with persistent Cu–O and Cu–O–C signals, signifying a fully passivated and chemically stable surface.

The morphological evolution of Cu_3_(HHTP)_2_ on the CuSCN film was analyzed using AFM and FESEM. As shown in Figure 5a, the pristine CuSCN film is composed of densely packed CuSCN particles, with an average roughness (*S*_a_) of merely 1.60 nm as measured by AFM topography. After the reactive dipping, the surface morphology changed significantly due to the growth of MOF bumps on the CuSCN film. For CuSCN-0.5MOF (Figure 5b), Cu_3_(HHTP)_2_ MOF seed layer appears to form anisotropically on the CuSCN surface, resulting in an island-like distribution. This observation is consistent with the growth behavior previously reported by Zheng et al.^51^ The nonuniform morphology contributes to an increase in the surface roughness (*S*_a_) to 4.06 nm. As the immersion time increases, we observed that the MOF continues to grow anisotropically on the CuSCN surface. Specifically, the newly formed MOF tends to grow preferentially on top of the existing MOF seeds, while areas of the CuSCN surface without initial MOF formation do not exhibit the obvious emergence of new seed layers. Due to this mode of MOF growth, the resulting morphology on the CuSCN surface ultimately resembles a Karst Fenglin-like landscape, characterized by numerous tall, isolated peaks standing on a relatively flat plain, as evidenced from the *S*_a_ of 14–15 nm (Figure 5c–e). FESEM images of the investigated samples echo AFM observations and are shown in Figure S5a–e. As depicted in Figure S5a, the pristine CuSCN film displays a densely packed morphology. For CuSCN-0.5MOF, noticeable changes in surface topology were observed, characterized by the gradual formation of discrete, evenly distributed MOF grains, as seen from Figure S5a,b. Further immersion for CuSCN-1MOF (Figure S5c) and CuSCN-2MOF (Figure S5d) leads to the formation of additional growth MOF structures on the existing MOF seeds rather than the exposed CuSCN surface. Notably, for CuSCN-4MOF­(Figure S5e), the surface achieves a Fenglin-like landscape with discrete MOF hills, indicating a unique Cu_3_(HHTP)_2_ MOF growth mode on CuSCN film (Figure S5a).

**Figure 5:**
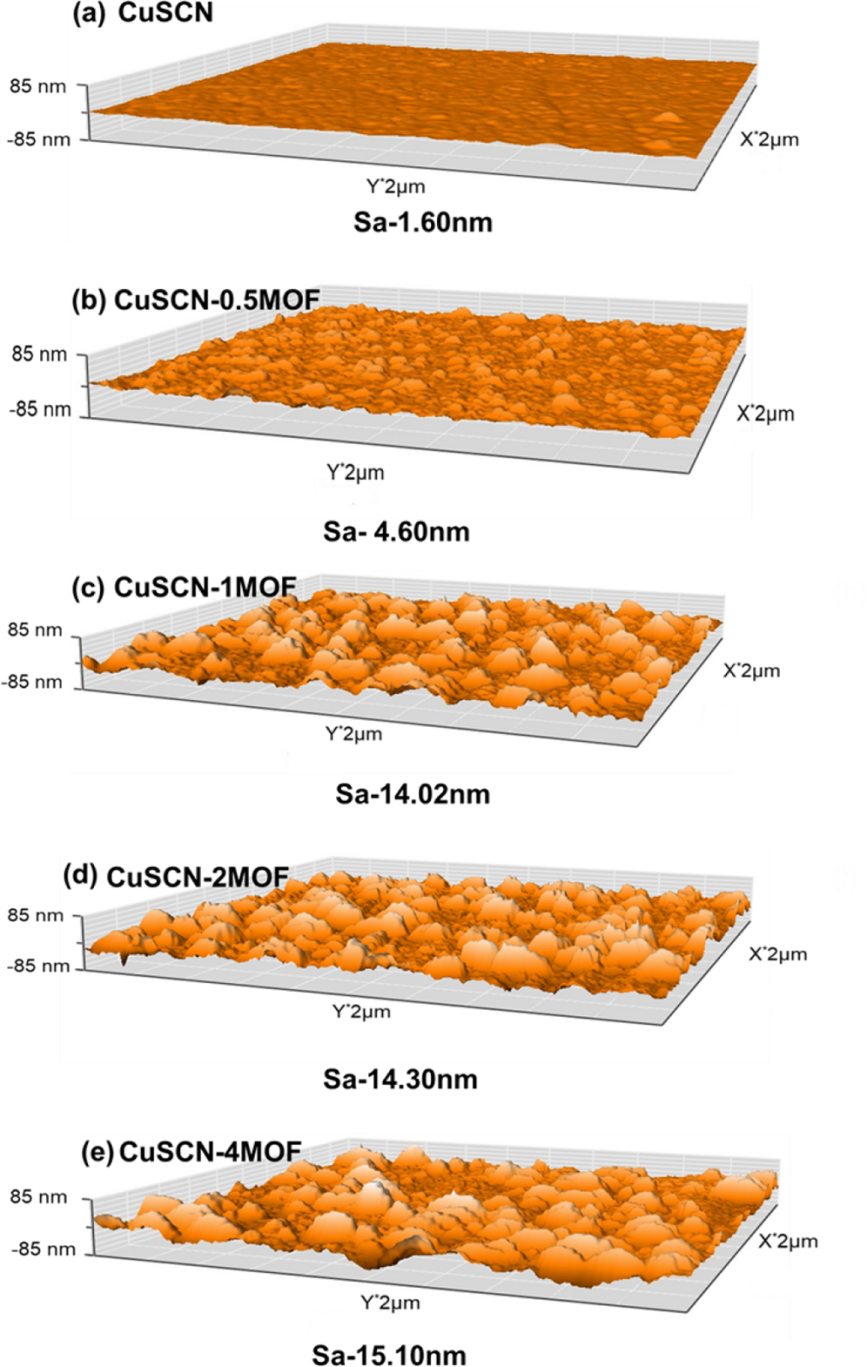
3D AFM images of (a) CuSCN, (b) CuSCN-0.5MOF, (c) CuSCN-1MOF, (d) CuSCN-2MOF, and (e) CuSCN-4MOF film on ITO substrate.

The cross-sectional FIB-FESEM images of the pristine CuSCN and MOF-modified CuSCN films are shown in Figure S6. The pristine CuSCN layer exhibits a compact and uniform morphology with an average thickness of ∼35 nm (Figure S6a). After dipping the films in the HHTP solution, additional MOF particles form on the CuSCN surface. Owing to their island-type growth behavior, it is difficult to precisely determine the MOF thickness from cross-sectional analysis alone. Instead, the modification primarily manifests as the progressive appearance of discrete MOF particles on the CuSCN surface with increasing dipping time. For the CuSCN-0.5MOF sample, the formation of the MOF results in new granular features with an average size of ∼40 nm (Figure S6b, while the underlying CuSCN layer thickness remains nearly unchanged. When the dipping duration is increased to 1 min (CuSCN-1MOF, Figure S6c), the overall CuSCN thickness is still maintained; however, the MOF particles begin to coalesce, forming larger aggregates of around 60 nm. Further extending the immersion time to 2 and 4 min (CuSCN-2MOF and CuSCN-4MOF, Figure S6d,e, respectively) leads to the formation of randomly distributed MOF particles with sizes ranging from 70 to 80 nm. Notably, the thickness of the CuSCN base layer remains essentially constant throughout this process.

This Fenglin-like, conductive Cu_3_(HHTP)_2_ MOF framework plays a role analogous to that of the mesoporous TiO_2_ layer in conventional n–i–p architectures, enhancing the interfacial contact and adhesion between CuSCN and PVSK. Based on the above characterizations, an imaginary mechanism of Fenglin-like Cu_3_(HHTP)_2_ MOF growth on CuSCN films is schematically illustrated in Figure 6. Specifically, the entire MOF growth process proceeds through three distinct steps. In Stage 1, the HHTP ligand initiates MOF nucleation by coordinating with Cu^2+^ ions present in the initial CuSCN film, which were formed as a result of a Cu^2+^-amine complexation, this triggers the formation of MOF nuclei in the early period of dipping. As the dipping time prolongs (Stage 2), more Cu^2+^ ions migrate or diffuse from the CuSCN thin film into the ligand solution. These Cu^2+^ ions interact with the ligand molecules and preferentially deposited onto the existing MOF nuclei, facilitating further MOF growth. This observation is supported by SEM and AFM analyses, where continuous growth of the MOF seeds instead of new MOF seeds formation was observed.

**Figure 6:**
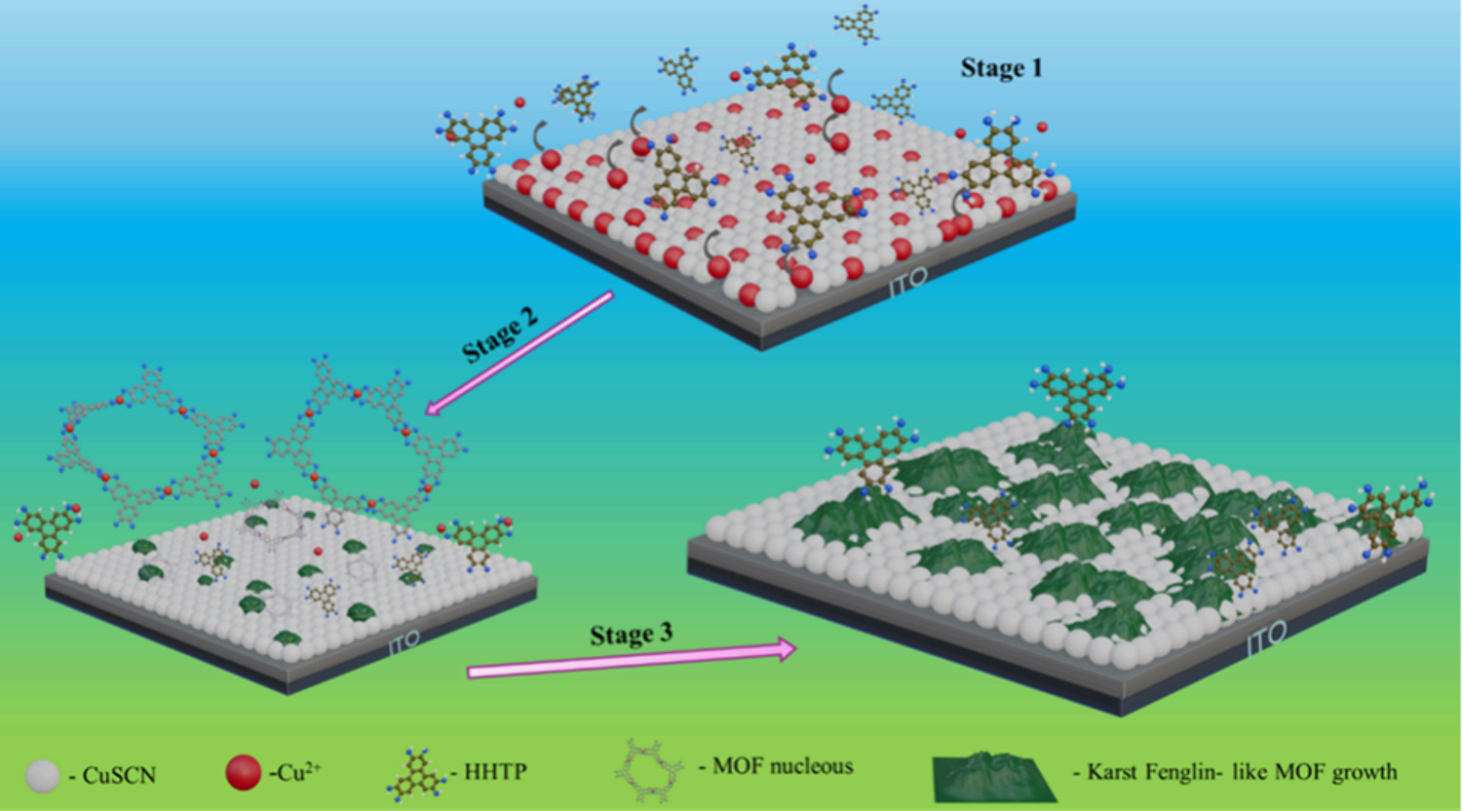
Schematic representation of direct MOF formation on CuSCN film.

GIXRD (Figure 1b) reveals an increase in the 27.2° peak intensity, further confirming MOF growth during this period. In Stage 3, when the dipping time is further extended, nearly all Cu^2+^ ions are consumed for MOF growth. Further increase in dipping time lead to the accumulation of free HHTP molecules on the thin film surface, as evidenced by the lack of additional MOF growth in SEM and AFM analyses.

### 3.2 PVSK Film Deposited on Cu_3_(HHTP)_2_/CuSCN Hybrid HTM

FESEM topographical images of PVSK films deposited on pristine and Cu_3_(HHTP)_2_-modified CuSCN layers are collected in Figure 7. For comparison, PVSK films deposited on bare ITO glass (PVSK/ITO) using the same deposition process are also included (Figure 7a). Due to the hydrophobic nature of the CuSCN surface compared to the bare ITO glass surface, the grain size of PVSK deposited on the CuSCN surface is larger.^52–8^ In addition, the surface PVSK films deposited on pristine CuSCN contain pinholes that randomly appear, as highlighted in Figure 7b. These pinholes could potentially create short-circuit pathways. Interestingly, the pinholes vanished in all Cu_3_(HHTP)_2_-modified CuSCN samples (Figure 7c–f). This improvement can be attributed to the rough surface of the MOF-modified CuSCN, which enhances adhesion at the CuSCN-PVSK interface and improves film uniformity. To further elucidate the interfacial structure, cross-sectional FESEM analysis was performed on CuSCN- and CuSCN-2MOF-based PSCs (Figure S7a,b). Both devices exhibit a similar PVSK thickness of ∼520 nm, confirming that MOF incorporation does not affect bulk PVSK film growth. Importantly, discrete MOF particles with an average size of ∼70 nm are clearly observed at the PVSK/CuSCN interface (Figure S7b), forming a discontinuous karst-like interlayer rather than a continuous film. This discrete interfacial architecture effectively improves physical contact and adhesion between the PVSK and CuSCN layers while avoiding the formation of a thick insulating barrier. As a result, efficient hole extraction is maintained, and interfacial nonradiative recombination at the HTM/PVSK interface is effectively suppressed.

**Figure 7:**
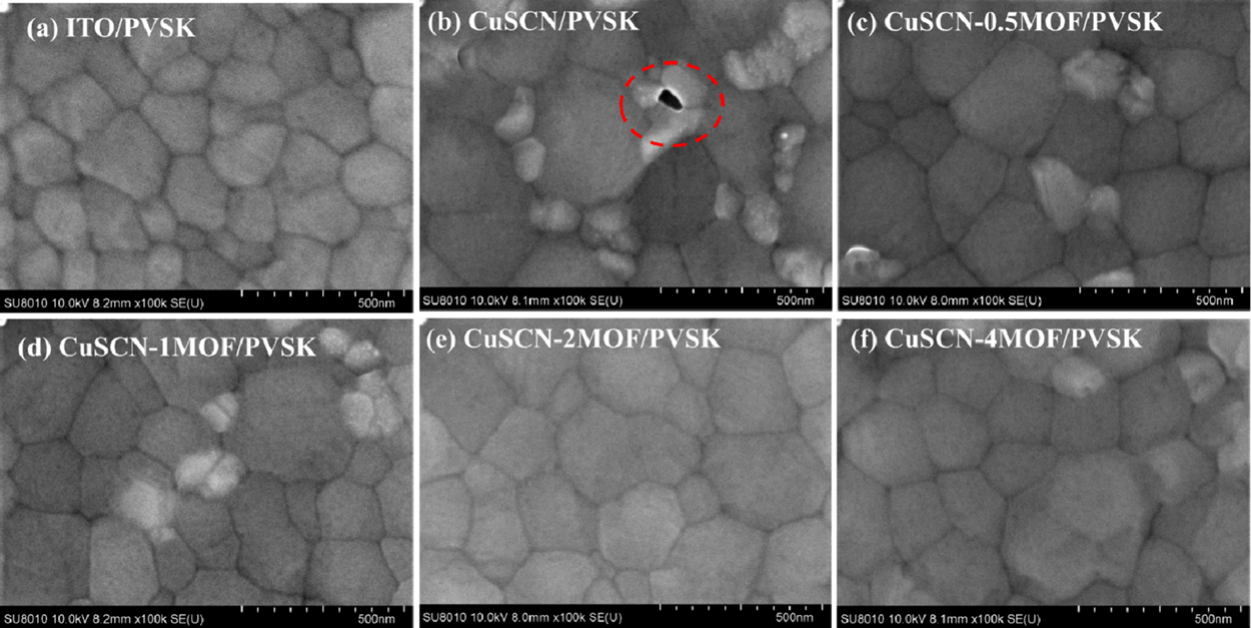
SEM of PVSK films on ITO and various CuSCN-based HTMs. (a) ITO/PVSK, (b) PVSK/CuSCN, (c) PVSK/CuSCN-0.5MOF, (d) PVSK/CuSCN-1MOF, (e) PVSK/CuSCN-2MOF, and (f) PVSK/CuSCN-4MOF.

XRD analysis was performed to investigate the phase composition and stability of PVSK films deposited on pristine and Cu_3_(HHTP)_2_-modified CuSCN surfaces, as shown in Figure 8. The XRD pattern of the PVSK film deposited on ITO exhibited no detectable PbI_2_ residue, indicating the initial high quality of the PVSK layer. However, when deposited on the pristine CuSCN surface, the same PVSK film displayed a distinct PbI_2_ peak at 12.7° (Figure 8a), corresponding to the (001) plane of PbI_2_.

**Figure 8:**
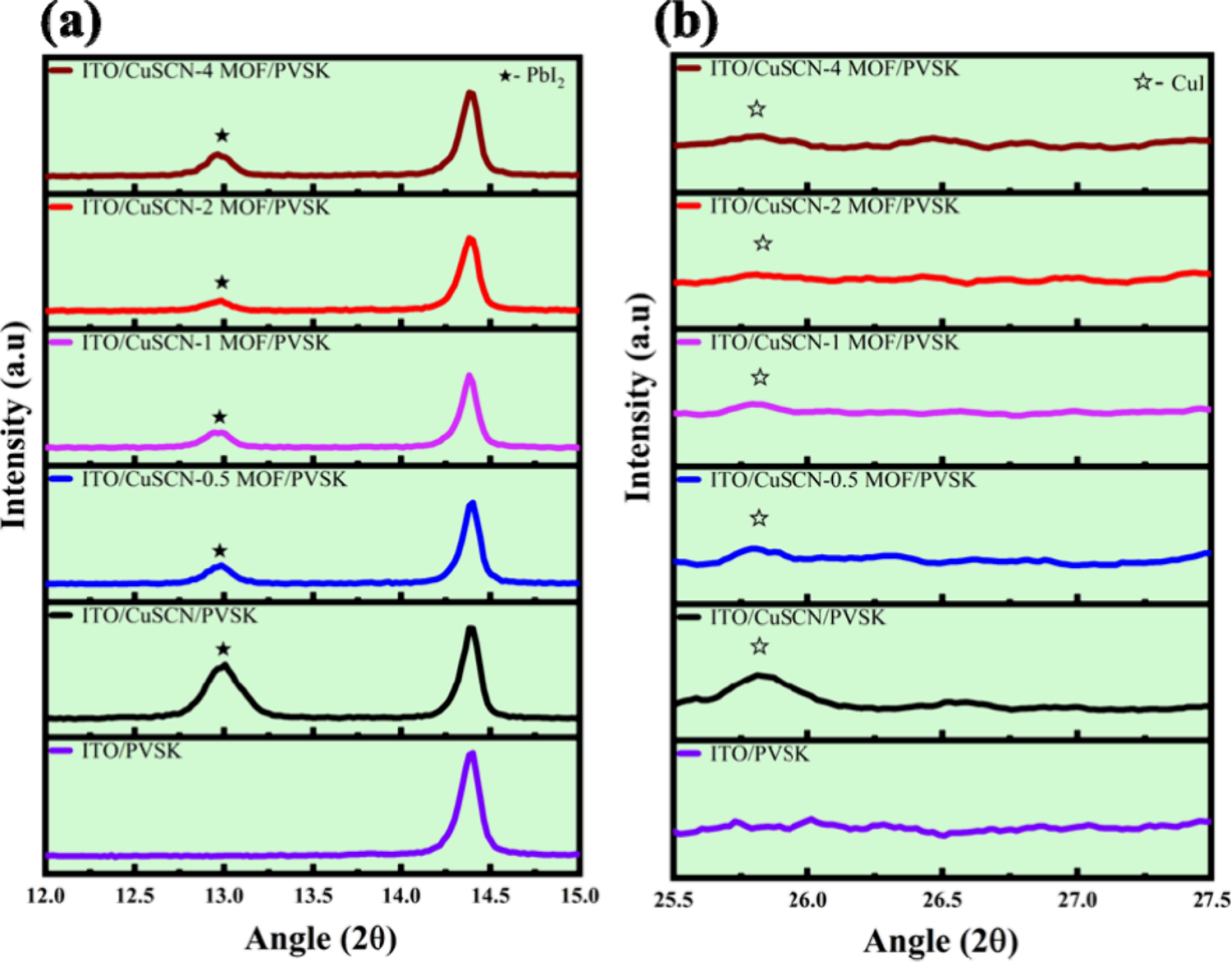
XRD patterns of PVSK films on ITO and various Cu_3_(HHTP)_2_/CuSCN HTMs, (a) at 2θ from 12 to 15° and (b) at 2θ from 25.5 to 27.5°.

This observation is attributed to the reactive degradation of the PVSK when it is in contact with CuSCN. Additionally, a new peak appeared at 25.5° (Figure 8b), which can be attributed to the (111) plane of CuI,^36,54^ suggesting the following interfacial reaction:

$${\mathrm{Cs}}_{x}{\mathrm{FA}}_{y}{\mathrm{MA}}_{\left(1-x-y\right)}\mathrm{Pb}(I_{1-z}{\mathrm{Br}}_{z}{)}_{3}+\mathrm{CuSCN}={\mathrm{Cs}}_{x}{\mathrm{FA}}_{y}{\mathrm{MA}}_{\left(1-x-y\right)}{\mathrm{Br}}_{z}\mathrm{SCN}↑+\mathrm{CuI}+\mathrm{Pb}I_{2}$$

Importantly, in Cu_3_(HHTP)_2_-modified samples, the formation of PbI_2_ and CuI arising from interfacial reactions is considerably suppressed. The presence of the Fenglin-like Cu_3_(HHTP)_2_ MOF framework on CuSCN films enables multiple interfacial improvements, including enhanced interfacial contact, improved adhesion, and better PVSK film integrity, and significantly reduced interfacial reactivity. These synergistic effects are crucial for constructing CuSCN-based PSC with both high efficiency and long-term stability.

### 3.3 Photovoltaic Performance

Reverse scan of the best-performing *JV* curves of inverted PSCs equipping CuSCN and Cu_3_(HHTP)_2_-modified CuSCN HTMs are shown in Figure 9a, while the photovoltaic parameters are summarized in Table 2 with statistical distribution of different voltaic parameters provide in Figure S8 Under the same PVSK composition and device fabrication conditions, the best PCEs for devices using pristine CuSCN, CuSCN-0.5MOF, CuSCN-1MOF, CuSCN-2MOF, and CuSCN-4MOF were 16.10, 18.96, 19.39, 20.05, and 17.82%, respectively (the corresponding *JV* curves provided in Figure S9. The low PCE of the device using pristine CuSCN can be attributed to its lower open-circuit voltage (*V*_OC_) and fill factor (FF), highlighting the importance of PVSK film integrity and the reactive CuSCN/PVSK interface, which is evidenced in the above discussion. Notably, all devices employed MOF-modified CuSCN, except for CuSCN-4MOF, exhibited higher short-circuit current density (*J*_SC_) than the pristine CuSCN-based device (22.70 mA/cm^2^), indicating the hole extraction efficiency of the Cu_3_(HHTP)_2_/CuSCN framework was significantly enhanced. The EQE spectra of the control CuSCN and CuSCN-2MOF-based PSCs are shown in Figure S10. The integrated *J*_SC_ values derived from the EQE measurements show a consistent trend with those obtained from the *J*–*V* curves. As shown in Figure S10, the CuSCN-2MOF/PVSK device exhibits consistently higher EQE values across the visible spectrum compared to the pristine CuSCN/PVSK device. This improvement explains the enhanced current density observed in the MOF-modified device and supports the formation of a high-quality, defect-suppressed PVSK layer on the CuSCN-MOF surface, which facilitates more efficient charge extraction and collection.^34,55,56^

**Table 2:** Photovoltaic Parameters of Various CuSCN-Based HTMs

**device type**		***J***_**SC**_ **(mA/cm** ^**2**^ **)**	***V***_**OC**_ **(V)**	**FF**	**PCE (%)**
CuSCN/PVSK	champion	22.7	1.01	0.70	16.10
	average	22.4 ± 0.3	1.00 ± 0.01	0.70 ± 0.01	15.91 ± 0.3
CuSCN0.50MOF/PVSK	champion	23.2	1.06	0.76	18.96
	average	23.27 ± 0.3	1.06 ± 0.01	0.74 ± 0.02	18.04 ± 0.7
CuSCN-1MOF/PVSK	champion	23.56	1.06	0.77	19.39
	average	23.23 ± 0.3	1.06 ± 0.01	0.74 ± 0.02	18.48 ± 0.6
CuSCN-2MOF/PVSK	champion	23.63	1.06	0.80	20.05
	average	23.43 ± 0.4	1.06 ± 0.01	0.78 ± 0.02	19.13 ± 0.9
CuSCN-4MOF/PVSK	champion	22.41	1.06	0.75	17.82
	average	22.94 ± 0.5	1.04 ± 0.02	0.72 ± 0.02	17.33 ± 0.5

**Figure 9:**
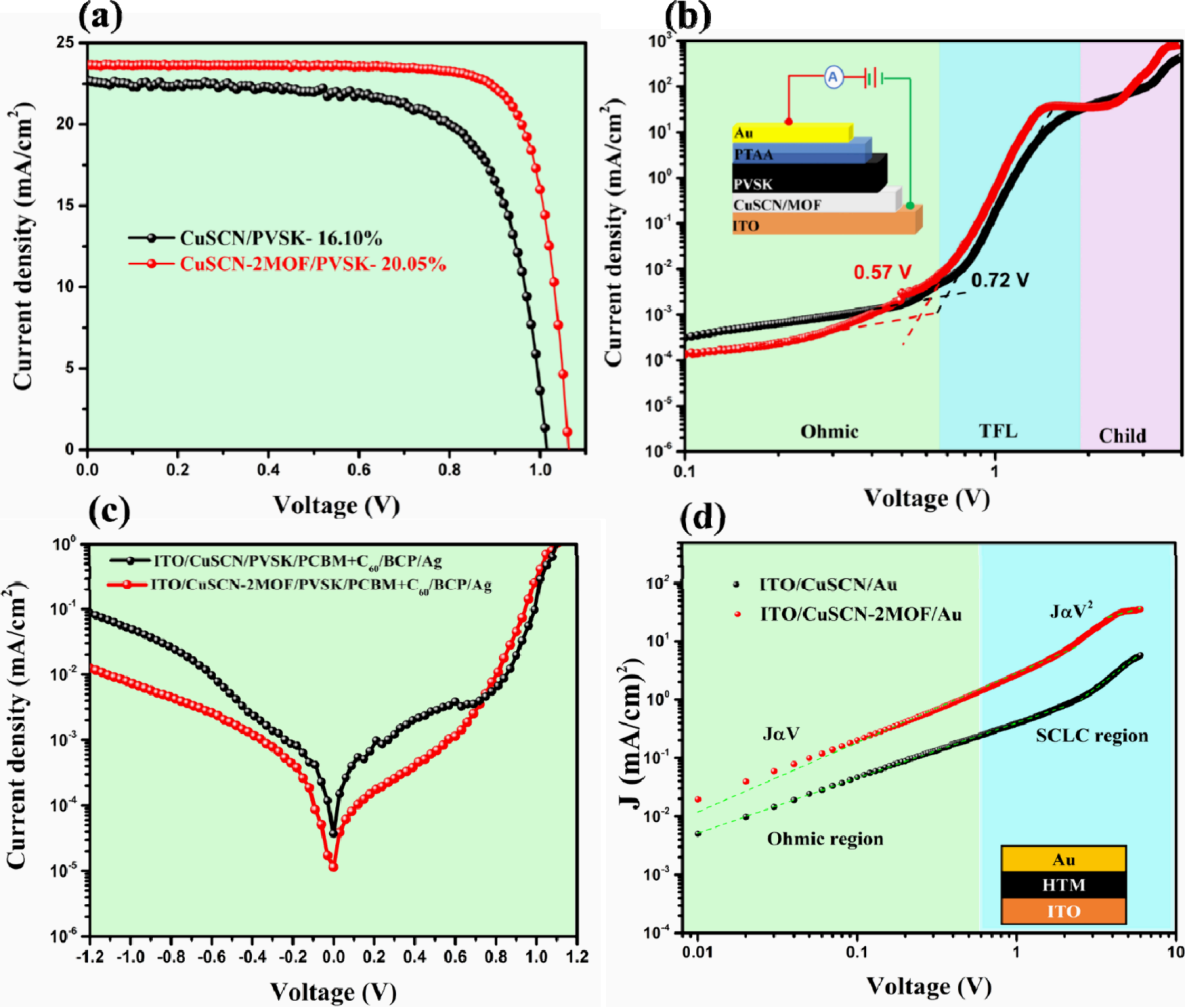
(a) *J*–*V* curves of the champion devices for CuSCN/PVSK and CuSCN-2MOF/PVSK-based PSCs (reverse scan, full *J*–*V* curves are provided in Figure S8). (b) Trap density measurement of CuSCN and CuSCN-MOF-based hole-only devices. (c) Dark *J*–*V* curves of CuSCN and CuSCN-MOF-based PSCs. (d) Hole mobility measurements of CuSCN and CuSCN-MOF-based films.

Furthermore, the FF of all Cu_3_(HHTP)_2_-modified CuSCN-based devices was significantly improved over that of the pristine CuSCN-based device, with CuSCN-2MOF achieving an FF of 0.80 compared to 0.70 for the pure CuSCN device. Since these devices share the same materials and fabrication conditions except the CuSCN/PVSK interface, the improvement must relate to the Fenglin-like MOFs, which were directly grown on CuSCN. The Fenglin-like MOFs create additional contact surface when compared with flat interfacial layers published elsewhere.^39–41,57^

Moreover, Cu_3_(HHTP)_2_ MOF is soft in nature, which can provide exceptional intimate contact with solution-processed PVSK film. Finally, the directly grown scenario greatly avoids the possible contact problems with bottom CuSCN during Cu_3_(HHTP)_2_ deposition. In the following discussion, we are going to demonstrate the improved interfacial properties between CuSCN and PVSK, which are all crucial for a highly efficient, highly stable CuSCN-based PSC. Furthermore, a comprehensive literature survey summarizing the state-of-the-art performance of CuSCN-based inverted PSC is presented in Table S2. The comparison clearly indicates that our MOF-modified CuSCN-based device exhibits performance comparable to the best-reported CuSCN-based PSCs to date.

To quantitatively assess the carrier trap density in CuSCN/PVSK and CuSCN-2MOF-based devices, the dark *J*–*V* measurements were performed on hole-only devices, as illustrated in Figure 9b. The recorded trap-filling limit voltage (*V*_TFL_) for CuSCN/PVSK and CuSCN-2MOF/PVSK-based PSCs was 0.72 and 0.57 V, respectively. The trap density (*n*_trap_) was then calculated using the equation:

$$n_{\mathrm{trap}}=\frac{2\varepsilon \varepsilon_{0}V_{\mathrm{TFL}}}{ed^{2}}$$

where *d* denotes the PVSK film thickness (480 nm), *e* is the elementary charge (1.66 × 10^–19^ C), ε represents the dielectric constant of PVSK film (28.8), and ε_0_ (8.854 × 10^–12^ F m^–1^) is the vacuum permittivity. Based on these parameters, the calculated trap densities were 8.85 × 10^15^ and 7.0 × 10^15^ cm^–3^ for CuSCN-based devices and CuSCN-2MOF-based devices, respectively. The lower *n*_trap_ observed in CuSCN-2MOF-based devices indicates that the newly formed Cu_3_(HHTP)_2_ in between the CuSCN/PVSK interface effectively reduces trap-assisted recombination at the interface. The Dark *J*–*V* curves of PSCs (Figure 9c) also revealed that the CuSCN-2MOF-based device exhibited a significantly reduced dark current density, approximately an order of magnitude lower than that of the device using pristine CuSCN. This substantial reduction is attributed to the suppression of nonradiative recombination, which facilitates efficient separation of photogenerated carriers, ultimately enhancing the overall photovoltaic performance of the device. The hole mobility of CuSCN and CuSCN-2MOF films was quantitatively assessed using the SCLC method on hole-only devices. The SCLC method applies the Mott–Gurney law, which relates current density (*J*) to the applied voltage (*v*), film thickness (*d*), and permittivity of the material (ε), and ε_0_ is the vacuum permittivity. The hole mobility (μ) according to the equation:^20,47,58^

$$J\left(v\right)=\frac{9}{8}\mu \varepsilon \varepsilon_{0}\frac{v^{2}}{d^{3}}$$

The result shown in Figure 9d reveals pristine CuSCN film exhibited a hole mobility of 0.020 cm^2^V^–1^s^–1^, which is consistent with previously reported values.^20,59^ The CuSCN-2MOF film displayed an improved hole mobility of 0.068 cm^2^V^–1^s^–1^. We believe that the enhanced hole mobility of CuSCN-2MOF is likely due to the improved film conductivity and more contact surface provided by the Fenglin-like landscape. The charge-extraction capability of CuSCN and CuSCN-2MOF HTMs was investigated using transient photocurrent (TPC) measurements. The TPC responses of the respective devices were recorded under identical illumination conditions, and the corresponding decay profiles are shown in Figure S11a. The CuSCN-based PSC exhibits a current decay lifetime of 4.79 μs, whereas the device employing the CuSCN-2MOF HTM shows a significantly faster decay of 1.18 μs. The substantially reduced decay time clearly indicates more efficient charge extraction in the CuSCN-2MOF-based device under the same operational conditions.

To further elucidate the role of the newly formed Fenglin-like CuSCN–MOF interfacial layer in promoting efficient charge extraction and transport, electrochemical impedance spectroscopy (EIS) measurements were performed (Figure S11b). The Nyquist plots of the CuSCN/PVSK and CuSCN–2MOF/PVSK devices were fitted using the equivalent circuit shown in the inset of Figure S11b. Both devices exhibit nearly identical series resistance values (*R*_1_ ≈ 13 Ω), confirming that the incorporation of the conductive Cu_3_(HHTP)_2_ MOF does not introduce an insulating barrier and preserves effective hole-transport characteristics at the interface. Notably, a pronounced enhancement in recombination resistance (*R*_2_) is observed, increasing from 692 Ω for the CuSCN/PVSK device to 1058 Ω for the CuSCN-2MOF/PVSK device (Table S2). This substantial increase in R_2_ indicates effective suppression of interfacial recombination and improved carrier dynamics at the hybrid interface.^55,60,61^ The Mott–Schottky (M–S) analysis (Figure S11c) was conducted to evaluate the built-in potential (*V*_bi_) of the PSCs. The CuSCN-2MOF-based device exhibits a higher *V*_bi_ of 1.09 V compared to 1.0 V for the pristine CuSCN-based device. The increased *V*_bi_ indicates more favorable energy-level alignment at the HTM/PVSK interface, which strengthens the internal electric field across the junction. This enhanced interfacial energetics promotes more efficient charge separation and hole extraction, suppresses interfacial recombination, and facilitates carrier transport, thereby contributing to the improved *V*_OC_ of the device.^55,60^

The Tauc plot in Figure S12a reveals the optical bandgap ∼3.85 eV of the CuSCN films slightly changed to the 3.73 eV value after HHTP ligand treatment. To elucidate the impact of HHTP treatment on the band alignment of the CuSCN layer, the valence band maximum (VBM) and conduction band maximum (CBM) of each film were determined by combining UPS and UV–visible spectroscopy data. The VBM was calculated using the following equation:^62,63^

$$\mathrm{VBM}=h\upsilon -(E_{\mathrm{cutoff}}-E_{\mathrm{onset}})$$

where *h*υ represents the incident photon energy (21.22 eV), *E*_cutoff_ corresponds to the high binding energy cutoff in the UPS spectrum, and *E*_onset_ signifies the low-energy onset of the valence band edge. The extracted *E*_cutoff_ values for CuSCN and CuSCN-2MOF films are 16.27 and 16.28 eV, respectively (Figure S12b). Similarly, the corresponding *E*_onset_ values are 0.45 and 0.80 eV for CuSCN and CuSCN-2MOF, respectively (Figure S12c). The CuSCN-2MOF film exhibits a higher VBM position of −5.73 eV compared to the pristine CuSCN film (−5.40 eV), which facilitates better hole injection into the PVSK with VBM of −5.78 eV (the corresponding UPS spectra of PVSK are provided in Figure S12d,e.

The corresponding energy band diagram is thus summarized in Figure 10a. As depicted, the VBM of CuSCN shifts from −5.40 to −5.73 eV upon MOF modification. Consequently, the valence band offset (Δ*E*_v_), defined as Δ*E*_v_ = VBM_PVSK_ – VBM_HTM_, is significantly reduced from 0.38 eV (pristine CuSCN) to 0.05 eV (CuSCN-MOF). This ∼330 meV reduction in energy barrier enhances hole extraction efficiency at the PVSK/HTM interface. Improved band alignment suppresses interfacial charge accumulation and recombination losses, thereby promoting better carrier selectivity and extraction. This favorable interfacial energetics resulted in improved *V*_OC_ and FF of CuSCN-MOF-based PSC.

**Figure 10:**
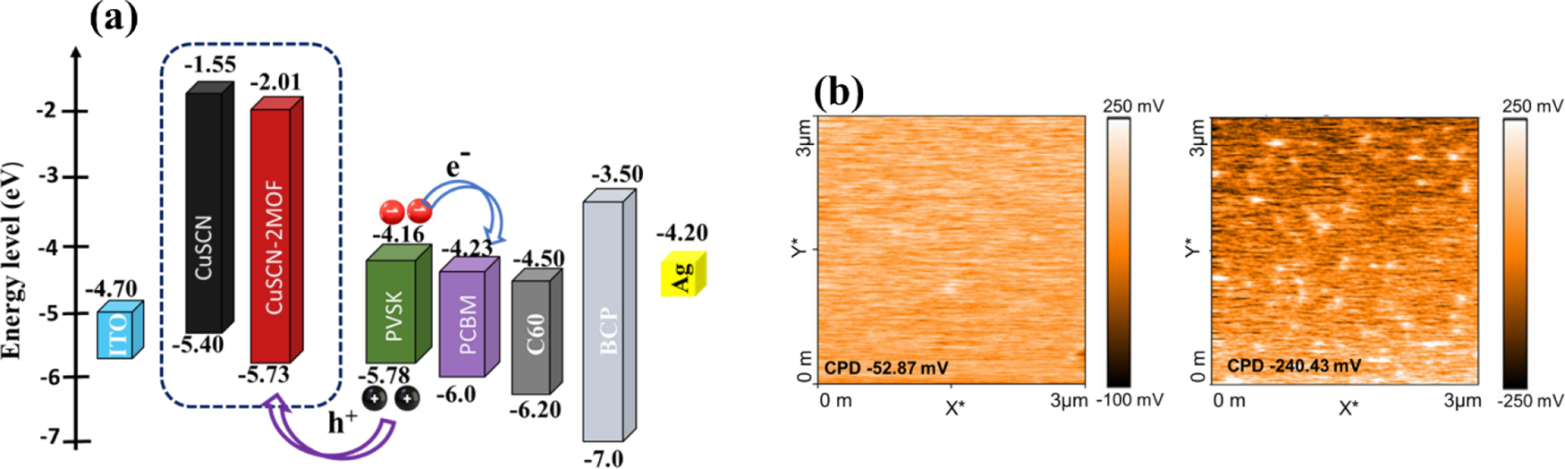
(a) Energy level diagram of the PSC. (b) KPFM analysis of CuSCN and CuSCN-2MOF films.

Additionally, the average surface CPD (contact potential difference) (Figure 10b) of the pristine CuSCN film was −52.87 mV, and the CPD of CuSCN-2MOF greatly decreased to −240.3 mV. Based on the CPD data, the surface work function of the pristine CuSCN film was determined to be 5.28 eV, while that of the CuSCN-2MOF film increased to 5.47 eV. This increase in work function suggests that MOF modification induces a downshifted Fermi level to the CuSCN film, thereby modulating its electronic band structure for enhanced charge carrier extraction and improved charge transport.^27,64,65^

SSPL measurements were conducted, as shown in Figure 11a. The zoomed-in SSPL spectra of CuSCN and CuSCN-2MOF samples reveal that CuSCN-2MOF exhibits better PL quenching, highlighting its superior hole extraction properties. In addition to SSPL, TRPL measurements were used to study the hole extraction dynamics of CuSCN and CuSCN-2MOF-based HTMs. The TRPL curves were fitted using the following biexponential decay equation.^18,20^

$$(t)=A_{1}e^{-t/\tau_{1}}+A_{2}e^{-t/\tau_{2}}+I_{0}$$

In this equation, *A*_1_ and *A*_2_ are pre-exponential factors corresponding to the two decay components: the fast decay term τ_1_, attributed to charge carrier transfer across the PVSK/HTM interface, and the slow decay term τ_2_, which represents bulk radiative recombination within the PVSK layer.^53,64,66,67^ The extracted parameters for the three film configurations are summarized in Table S3, and the fitted TRPL curves are shown in Figure 11b–d.

**Figure 11:**
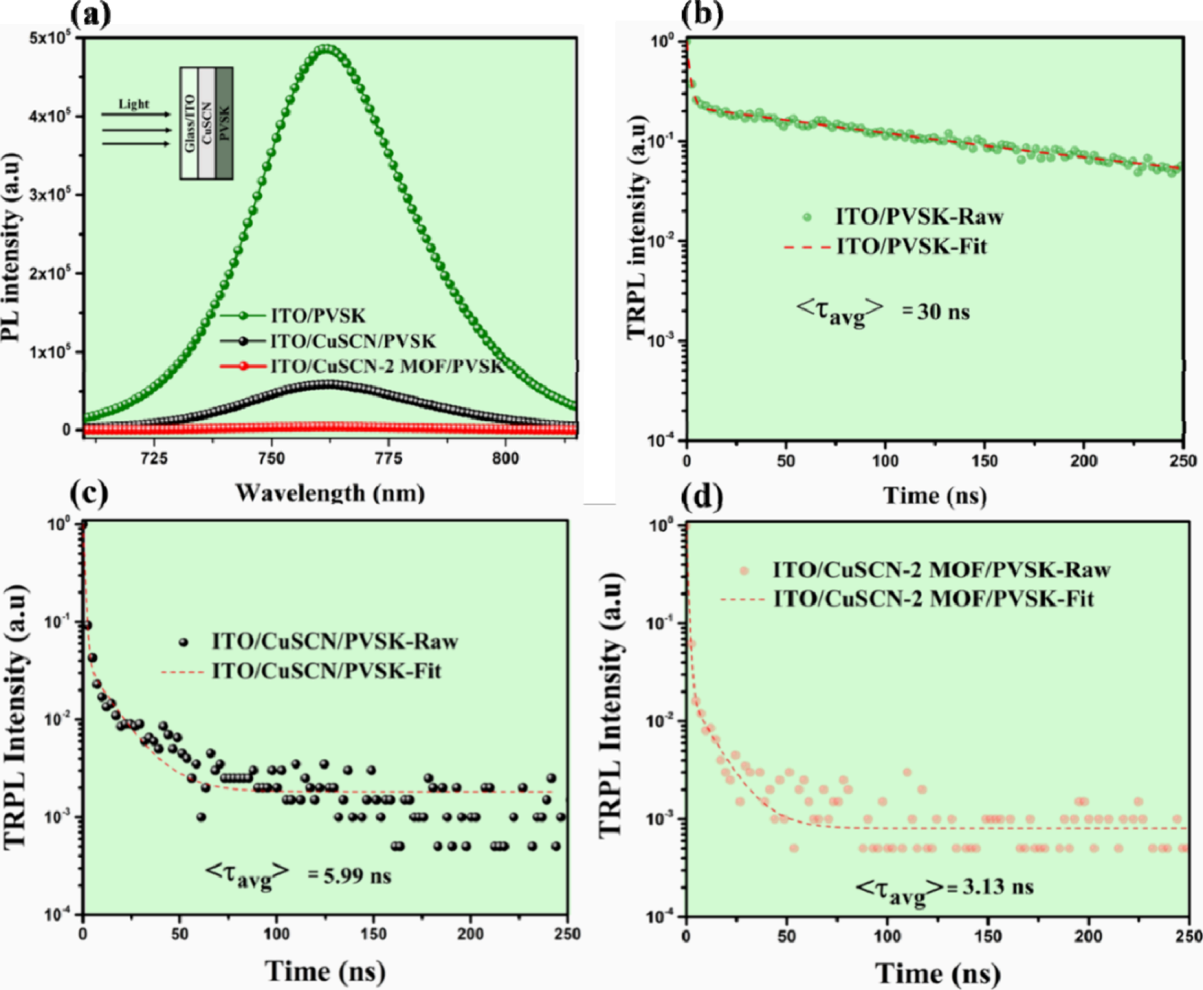
(a) SSPL of ITO/PVSK, ITO/CuSCN/PVSK, and ITO/CuSCN-2MOF/PVSK, (b) TRPL of ITO/PVSK, (c) TRPL of ITO/CuSCN/PVSK, (d) TRPL of ITO/CuSCN-2MOF/PVSK, respectively.

For the ITO/PVSK film (without HTM), the fast decay time τ_1_ is 5.16 ns, and the slow decay component τ_2_ is 150.01 ns. The long τ_2_ indicates minimal interfacial charge extraction and dominant radiative recombination within the bulk PVSK. The corresponding average carrier lifetime (⟨τ_avg_⟩)^68^

$$\langle \tau_{\mathrm{avg}}\rangle =\frac{A_{1}\tau_{1}+A_{2}\tau_{2}}{A_{1}+A_{2}}$$

is 30.00 ns, reflecting inefficient hole extraction at the PVSK surface (Figure 11b). In contrast, the ITO/CuSCN/PVSK (Figure 11c) film shows a slightly shorter τ_1_ of 4.97 ns and a significantly reduced τ_2_ of 13.47 ns. This reduction in both time constants suggests enhanced interfacial charge extraction and suppressed radiative recombination due to the incorporation of the CuSCN HTM. As a result, the ⟨τ_avg_⟩ drops substantially to 6.0 ns, indicating improved charge separation and transport at the HTM interface. The most pronounced improvement is observed in the ITO/CuSCN-2MOF/PVSK film. Here, τ_1_ is further reduced to 2.87 ns, and τ_2_ decreases to 11.27 ns. The notably short τ_1_ reflects rapid hole extraction facilitated by the modified CuSCN-2MOF interface, while the reduced τ_2_ suggests minimized recombination losses in the PVSK bulk. Consequently, the ⟨τ_avg_⟩ is significantly lowered to 3.10 ns, confirming the superior interfacial quality and efficient hole transfer enabled by the Cu_3_(HHTP)_2_-CuSCN HTM layer.

The ambient stability of unencapsulated CuSCN and CuSCN-2MOF-based PSCs was first evaluated under controlled storage conditions (15 ± 5% RH, 25 ± 1 °C) in the dark. The evolution of PCE was monitored for up to 700 h. As shown in Figure 12a, the pristine CuSCN-based device exhibits a gradual performance decay, retaining approximately 60% of its initial PCE after 700 h. In contrast, the CuSCN-2MOF-based device demonstrates markedly improved stability, maintaining ∼ 82% of its initial PCE under identical conditions, underscoring the stabilizing effect of Cu_3_(HHTP)_2_-modified CuSCN. In addition to PCE retention, a pronounced difference is observed in the temporal evolution of the FF (Figure S13c). The FF of the pristine CuSCN-based PSC decreases to 71% of its initial value, whereas the CuSCN-2MOF-based device retains 89% of its initial FF. The superior FF preservation is attributed to effective stabilization of the HTM/PVSK interface enabled by the Fenglin-like Cu_3_(HHTP)_2_ MOF interlayer, which mitigates interfacial degradation and maintains efficient charge transport pathways.

**Figure 12:**
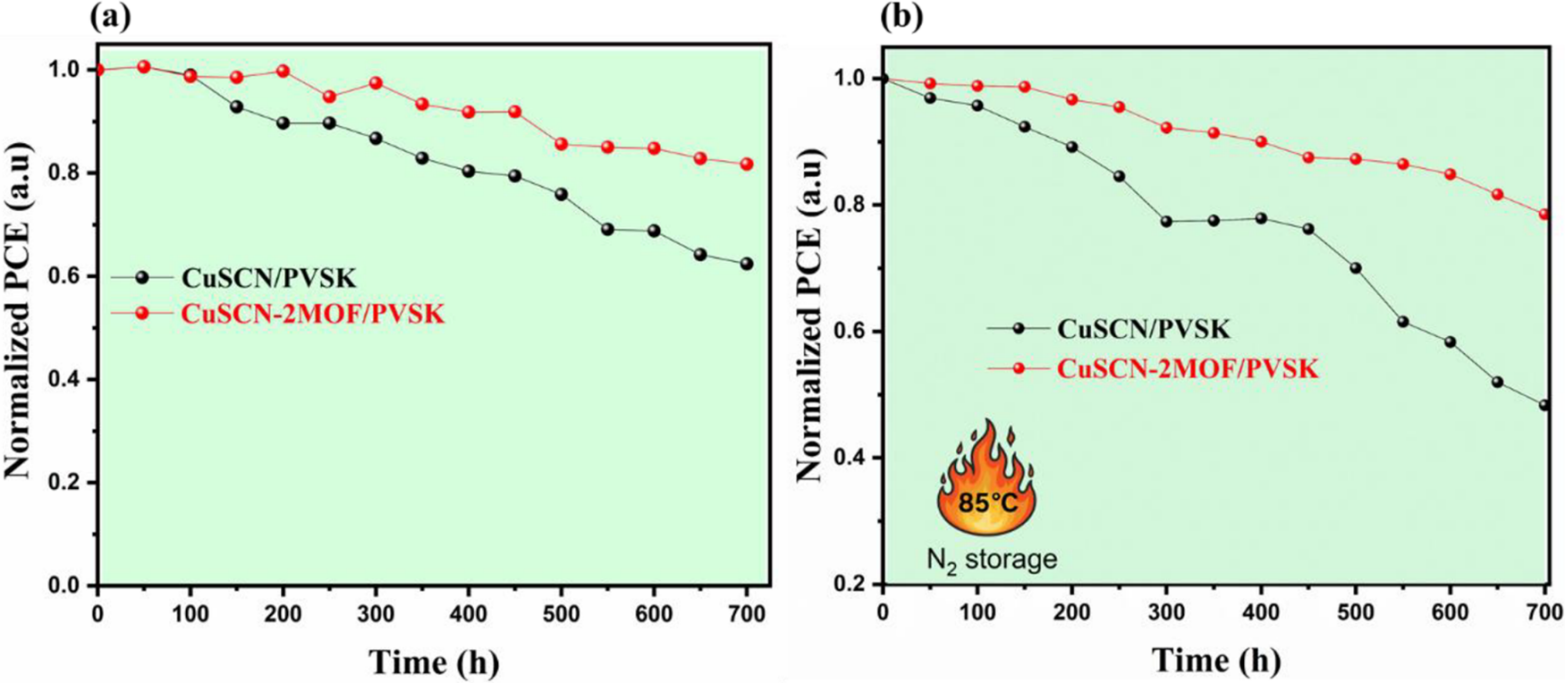
Stability of CuSCN- and CuSCN-2MOF-based PSCs under (a) ambient storage (15 ± 5% RH, 25 ± 1 °C) and (b) thermal aging at 85 °C in N_2_ without encapsulation.

Thermal stability was further assessed by storing unencapsulated devices at 85 °C in a N_2_-filled glovebox (Figure 12b). After 700 h of continuous thermal stress, the PCE of the pristine CuSCN-based device declines to ∼45% of its initial value. In contrast, the CuSCN-2MOF-based PSC retains ∼75% of its initial PCE, highlighting the enhanced thermal robustness of the HTM/PVSK interface achieved through MOF integration.

## 4 Conclusions

In summary, this study presents an innovative strategy for in situ formation of a thin, three-dimensional Fenglin-like Cu_3_(HHTP)_2_ MOF, directly on spin-coated CuSCN films. This hybrid HTM effectively addresses the interfacial reactivity and poor contact issues typically observed between CuSCN and the perovskite absorber layer. By precisely controlling the deposition conditions of the HHTP ligand, we achieved enhanced charge extraction, suppressed interfacial nonradiative recombination, and improved hole injection from the perovskite into the HTM layer. The resulting CuSCN/Cu_3_(HHTP)_2_-based PSC devices exhibited a significantly improved PCE of 20.05%, compared to 16.10% for devices with unmodified CuSCN. In addition to efficiency improvement, the modified devices exhibit superior operational stability, retaining 82% of their initial PCE after 700 h of ambient storage and ∼75% after 700 h of thermal aging at 85 °C under N_2_ storage, whereas pristine CuSCN-based devices retain only ∼60 and ∼45%, respectively. These findings clearly demonstrate the effectiveness of MOF interfacial modification in enhancing both device performance and operational stability. This work introduces a novel and scalable interface engineering approach that enables controlled MOF growth on CuSCN, offering a promising pathway for resolving scientific and technical limitations associated with these inorganic HTMs. Our results pave the way toward the practical development of low-cost, stable, and efficient perovskite solar cells incorporating CuSCN-based HTMs.

## Supplementary Material



## Notes

Raman spectra of CuSCN, Cu_3_(HHTP)_2_, and reference Cu_3_(HHTP)_2_ powders (Figure S1); photographs of CuSCN dissolved in NH_3_ and CuSCN–NH_3_ solutions with added HHTP (Figure S2); UV–vis absorption spectra of CuSCN films dipped in ethanol for 0.5, 1, 2, and 4 min and corresponding MOF solutions collected at different dipping times (Figure S3); photographs of CuSCN-NH_3_ solutions with HHTP, pristine CuSCN and CuSCN-MOF films on ITO, and transmittance spectra of CuSCN and CuSCN-MOF films on ITO (Figure S4); FESEM images of CuSCN, CuSCN-0.5MOF, CuSCN-1MOF, CuSCN-2MOF, and CuSCN-4MOF films on ITO substrates (Figures S5 and S6); cross-sectional FESEM images of CuSCN- and CuSCN-2MOF-based PSCs (Figure S7); statistical distributions of photovoltaic parameters for CuSCN- and CuSCN-MOF-based PSCs (Figure S8); *J*–*V* curves of PSCs employing different CuSCN-MOF HTMs under reverse and forward scans (Figure S9); literature survey of CuSCN-based p–i–n PSCs (Table S1); EQE spectra of CuSCN- and CuSCN-2MOF-based PSCs (Figure S10); TPC, EIS and Mott–Schottky measurements with corresponding fitted parameters (Figure S11 and Table S2); Tauc plots, *E*_cutoff_, and *E*_onset_ values of CuSCN, CuSCN-2MOF, and PVSK films (Figure S12); TRPL decay parameters for ITO/PVSK, ITO/CuSCN, and ITO/CuSCN-2MOF films (Table S3); and evolution of *J*_SC_, *V*_OC_, and FF of CuSCN- and CuSCN-2MOF-based PSCs during ambient storage and thermal aging at 85 °C under N_2_ atmosphere (Figure S13) (PDF)
