# Supplementary material for: Direct Formation of Karst Fenglin-like Cu-Based Metal–Organic Framework on CuSCN Hole Transport Layer for Highly Efficient and Stable Perovskite Solar Cells
Source: ACS Appl Mater Interfaces. 2026 May 12;18(20):28840–56. doi: 10.1021/acsami.6c06348 (PMC13220225; doi:10.1021/acsami.6c06348)
Supplement: Supplementary file 1 [file am6c06348_si_001.pdf]

Supporting Information

Direct Formation of Karst Fenglin-like Cu-based Metal-Organic Framework on CuSCN Hole Transport Layer for Highly Efficient and Stable Perovskite Solar Cells

*Nideesh Perumbalathodi[1], Sarin Sajjadu Krishna[1], Tzu-Sen Su[2], Tzu-Chien Wei[1,3]**

[1]Department of Chemical Engineering, National Tsing Hua University, 101, Section 2, Kuang Fu Road, Hsinchu 30013, Taiwan (R.O.C).

[2] Graduate Institute of Energy and Sustainability Tech, National Taiwan University of Science and Technology, No.43, Keelung Rd., Sec. 4, Da'an Dist., Taipei City 106335, Taiwan (R.O.C).

[3] Research Center for Critical Issues, Academia Sinica, South Campus, No 100, Section 1, Guiren 13th Road, Guiren District, Tainan City 711010, Taiwan (R.O.C).

E-mail: tcwei@mx.nthu.edu.tw

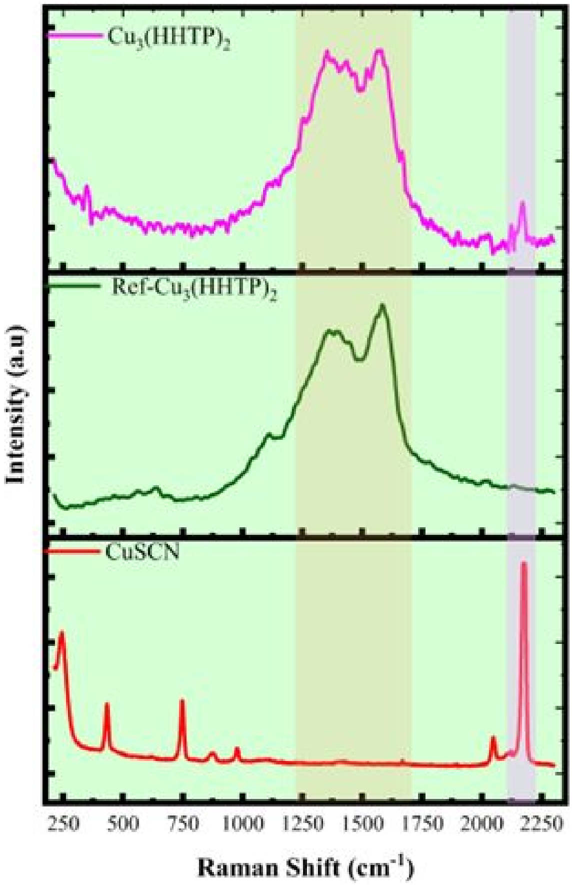

Figure S1. Raman spectra of CuSCN, Ref-Cu$_3$(HHTP)$_2$, and Cu$_3$(HHTP)$_2$ powders.

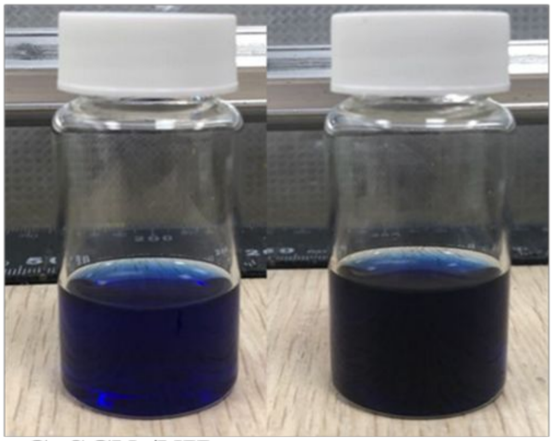

Figure S2. (a) Photograph of CuSCN dissolved in NH_3 and CuSCN-NH_3 solution with added HHTP.

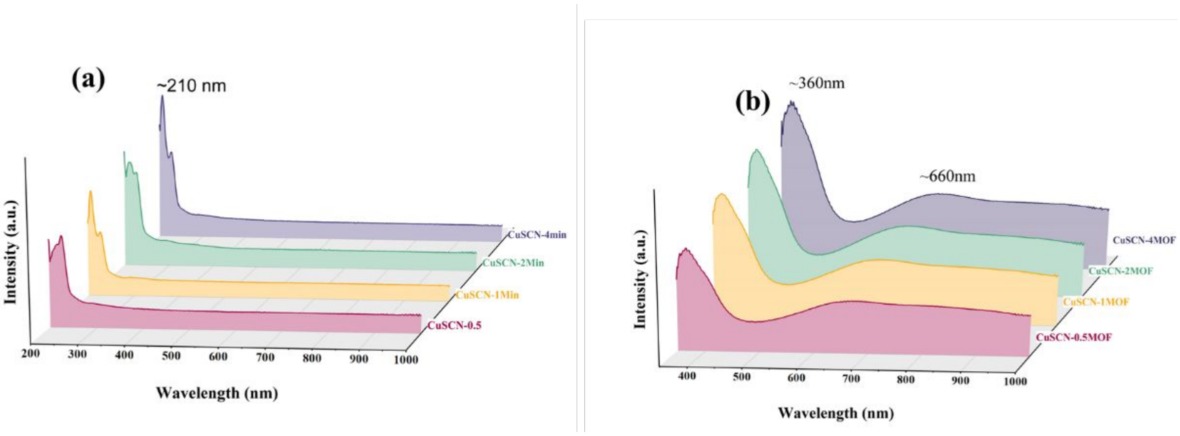

Figure S3. (a) UV of CuSCN film dipped in ethanol for 0.5,1,2 and 4 minutes (CuSCN-0.5, CuSCN-1Min, CuSCN-2Min, and CuSCN-4Min respectively), (b) UV of MOF solution collected at different dipping times: 0.5, 1, 2, and 4 minutes (CuSCN-0.5MOF, CuSCN-1MOF, CuSCN-2MOF and CuSCN-4MOF, respectively).

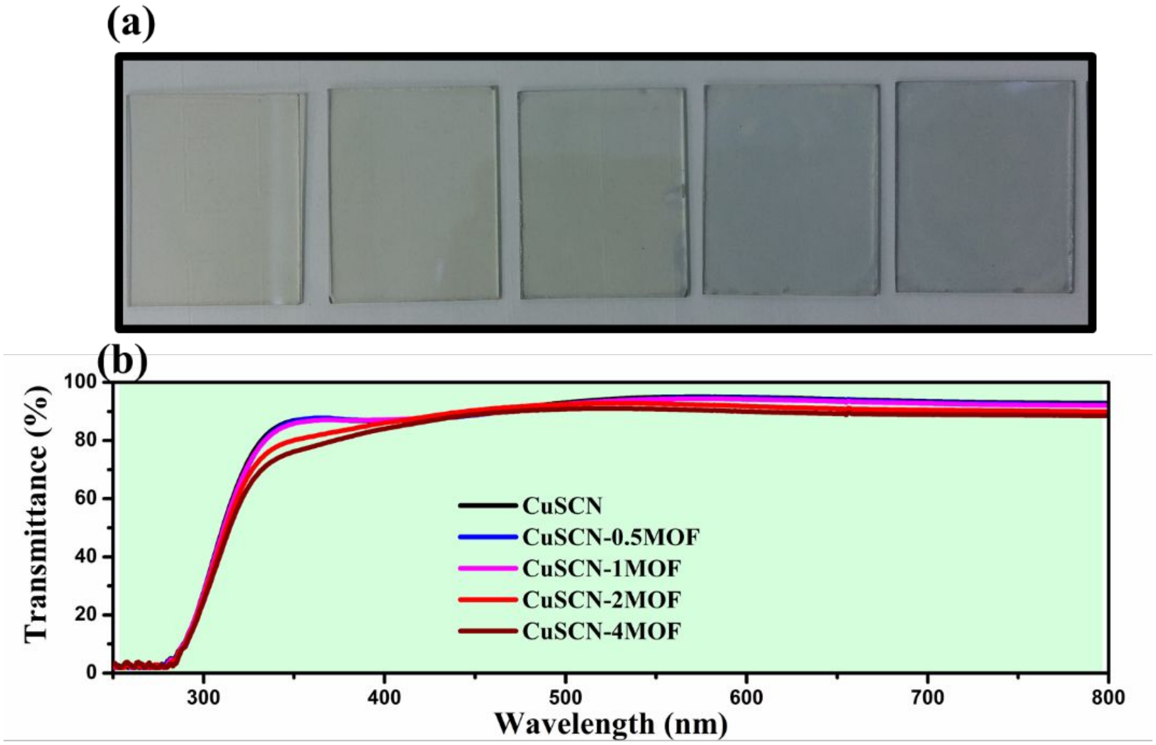

Figure S4. (a) Photograph of pristine CuSCN and various CuSCN-MOF films on ITO, (b) Transmittance spectra of CuSCN, CuSCN-0.5MOF, CuSCN-1MOF, CuSCN-2MOF, CuSCN-4MOF, respectively.

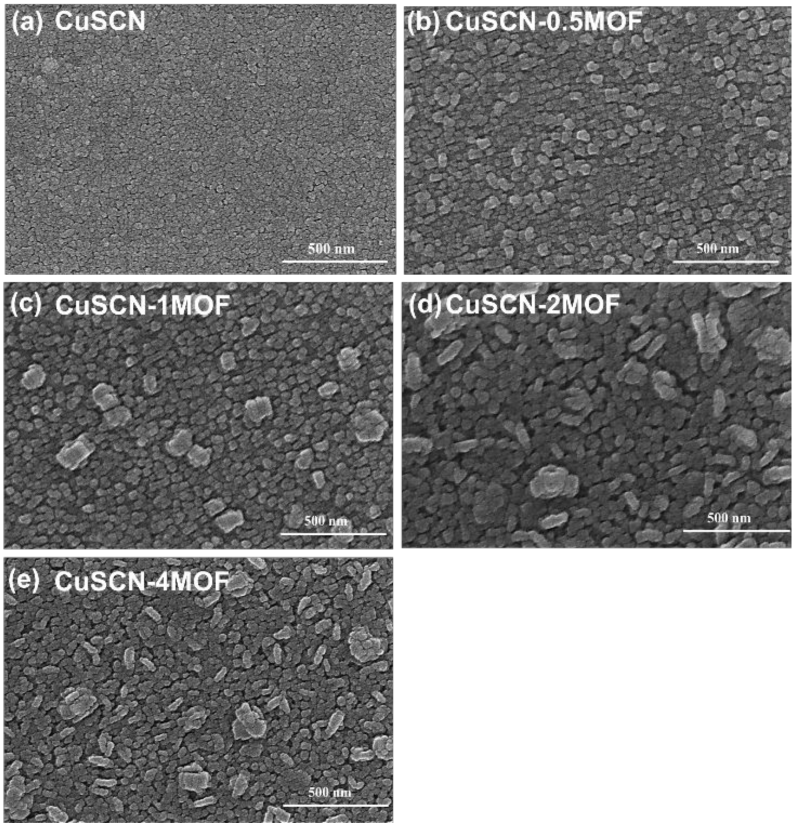

Figure S5. FESEM of (a) CuSCN, (b) CuSCN-0.5MOF, (c) CuSCN-1MOF, (d) CuSCN-2MOF, and (e) CuSCN-4MOF film on ITO substrate.

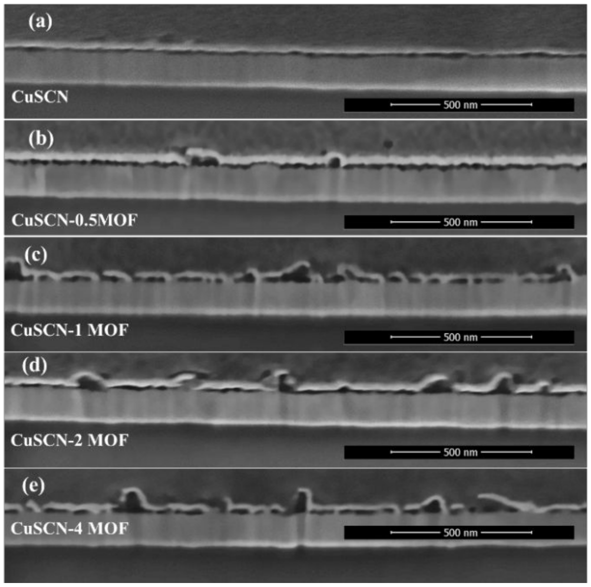

Figure S6. FESEM of (a) CuSCN, (b) CuSCN-0.5MOF, (c) CuSCN-1MOF, (d) CuSCN-2MOF, and (e) CuSCN-4MOF film on ITO substrate.

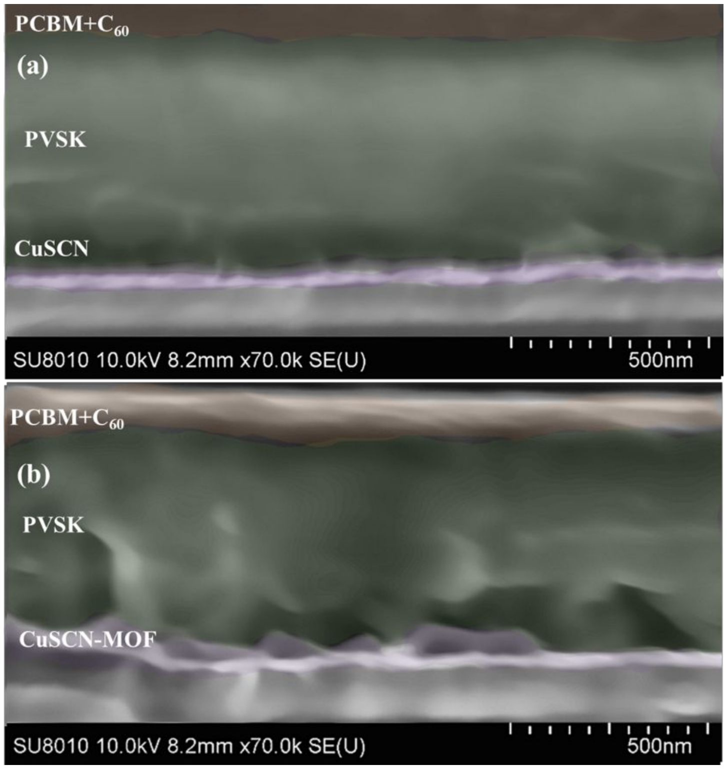

Figure S7. Cross-sectional FESEM images of (a) CuSCN and (b) CuSCN-2MOF-based PSCs.

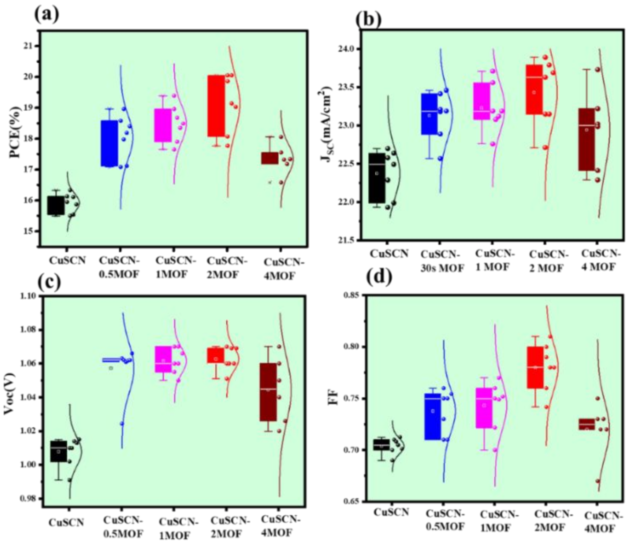

Figure S8. Statistical distribution of photovoltaic parameters: (a) PCE, (b) J_{SC}, (c) Voc, and (d) FF for CuSCN, CuSCN-0.5MOF, CuSCN-1MOF, CuSCN-2MOF, and CuSCN-4MOF, respectively.

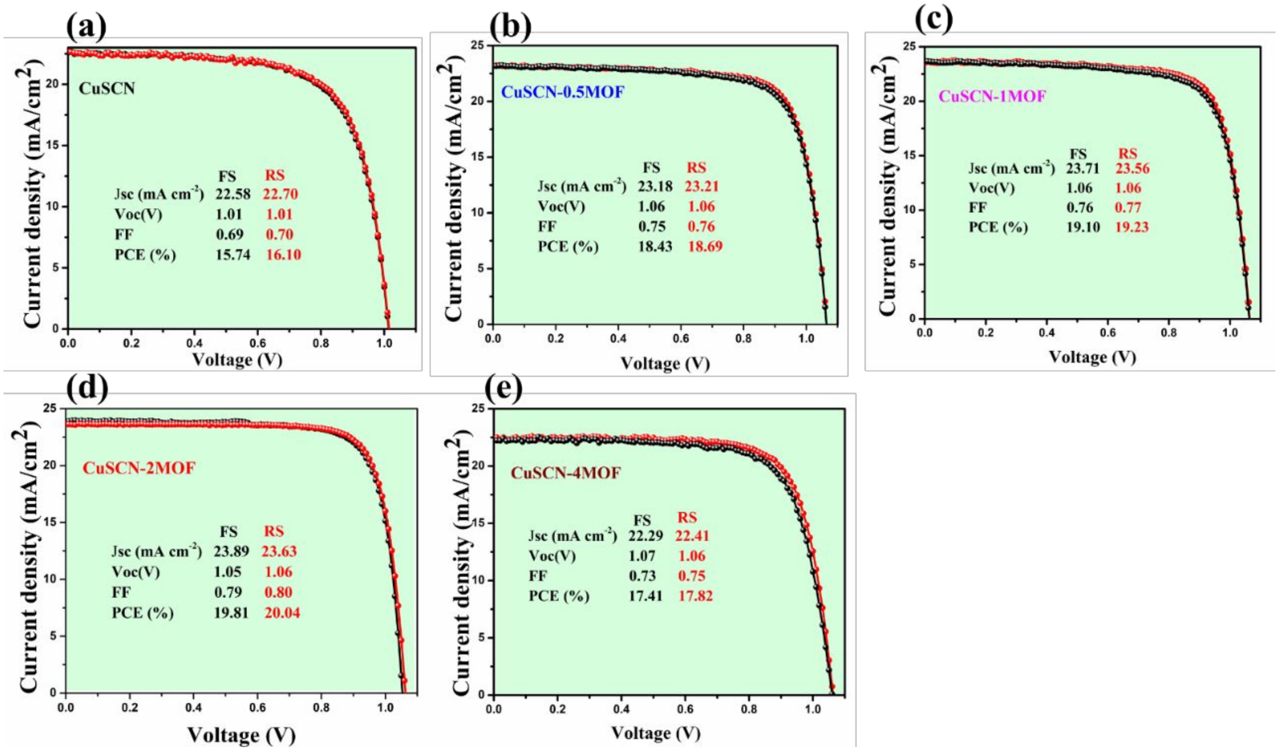

Figure S9. J-V curves of (a) CuSCN, (b) CuSCN-0.5MOF, (c) CuSCN-1MOF, (d) CuSCN-2MOF and (e) CuSCN-4MOF based PSC, respectively. (RS-Reverse scan, FS-Forward scan).

Device Structure	HTM	PVSK	Efficiency (%)	Year[reference]
**p-i-n**	CuSCN	$MAPbI_3$	10.8	2015[1]
	CuSCN	$MAPbI_3$	16.0	2015[2]
	CuSCN	$MAPbI_3$	16.60	2015[3]
	CuSCN(NH_3)	$MAPbI_3$	17.50	2017[4]
	CuSCN: CuI	$MAPbI_3$	18.80	2018[5]
	CuSCN:F_4TNCQ	$MAPbI_3$	15.0	2019[6]
	CuSCN/KSCN	$MAPbI_3$	14.9	2019[7]
	CuSCN: CuI	$MAPbI_3$	18.8	2021[8]
	CuSCN	$MAPbI_3$	19.19	2021[9]
	CuSCN	$(FAPbI_3)_{0.97}(MAPbI_3)_{0.03}$	18.71	2022[10]
	Cl_2-CuSCN	$MAPbI_3$	20.10	2022[11]
	CuSCN-2MOF	$Cs_{0.05}((FA_{0.84}MA_{0.16}))_{0.95}Pb(I_{0.84}Br_{0.16})_3$	20.05	**This work**

Table S1 -Literature survey of CuSCN-based p-i-n PSC

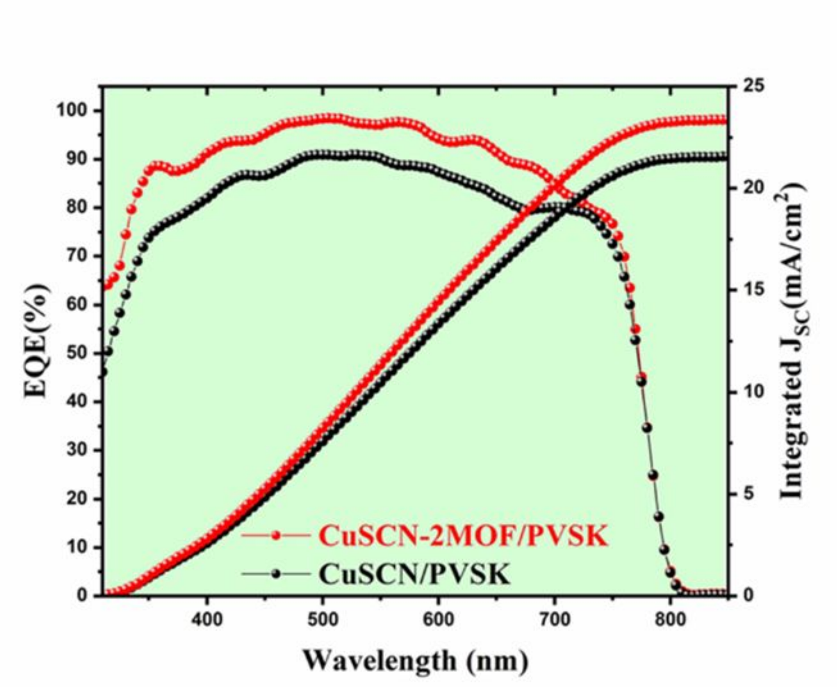

Figure S10. EQE spectra of CuSCN/PVSK and CuSCN-2MOF/PVSK-based PSC, respectively.

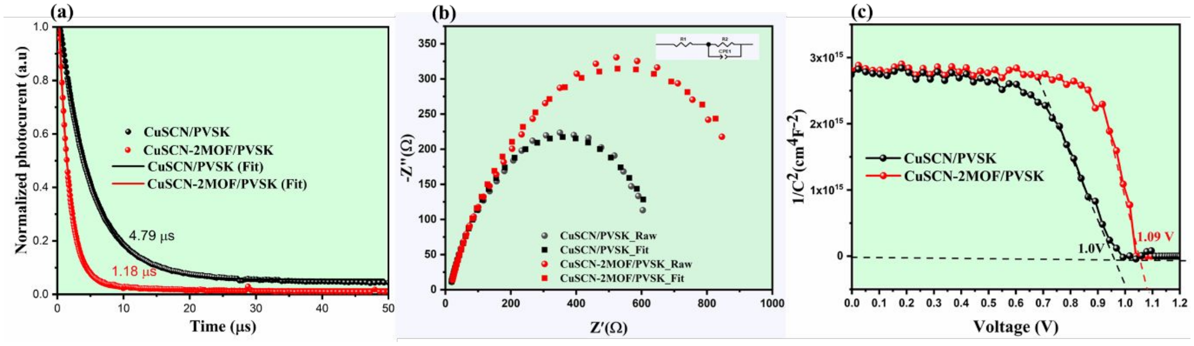

Figure S11. (a) TPC, (b) EIS, and (c) Mott–Schottky measurements of CuSCN/PVSK and CuSCN-2MOF/PVSK-based PSCs, respectively.

Table S2 EIS parameters of CuSCN/PVSK and CuSCN-2MOF/PVSK-based PSCs, respectively.

Device	R_1 (Ω)	R_2 (Ω)
CuSCN/PVSK	13.15	692
CuSCN–2MOF/PVSK	13.39	1058

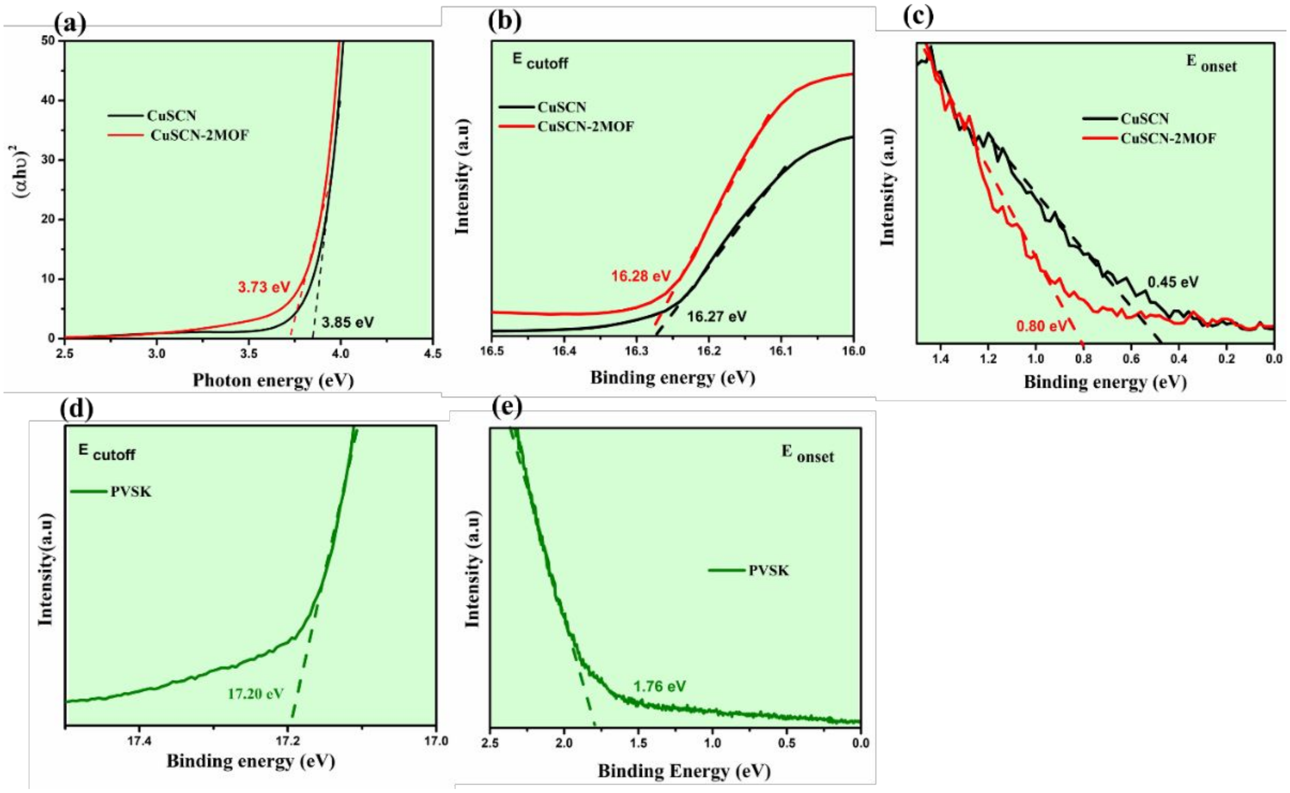

Figure S12. (a) Tauc plot, (b) E_{cutoff} and (c) E_{onset} of CuSCN and CuSCN-2MOF films, (d) E_{cutoff} and (e) E_{onset} of PVSK film.

Table S3. TRPL Decay parameter for ITO/PVSK, ITO/CuSCN/PVSK, and ITO/CuSCN-2MOF/PVSK films.

Film	A1	A2	τ_1	τ_2	τ_{avg}
ITO/PVSK	0.780	0.207	5.16 ± 0.22	150.0 ± 6.4	30.0 ± 1.5
ITO/CuSCN/PVSK	0.962	0.040	4.97 ± 0.18	13.5 ± 0.5	6.00 ± 0.3
ITO/CuSCN-2MOF/PVSK	0.984	0.020	2.87 ± 0.12	11.3 ±0.4	3.10 ± 0.2

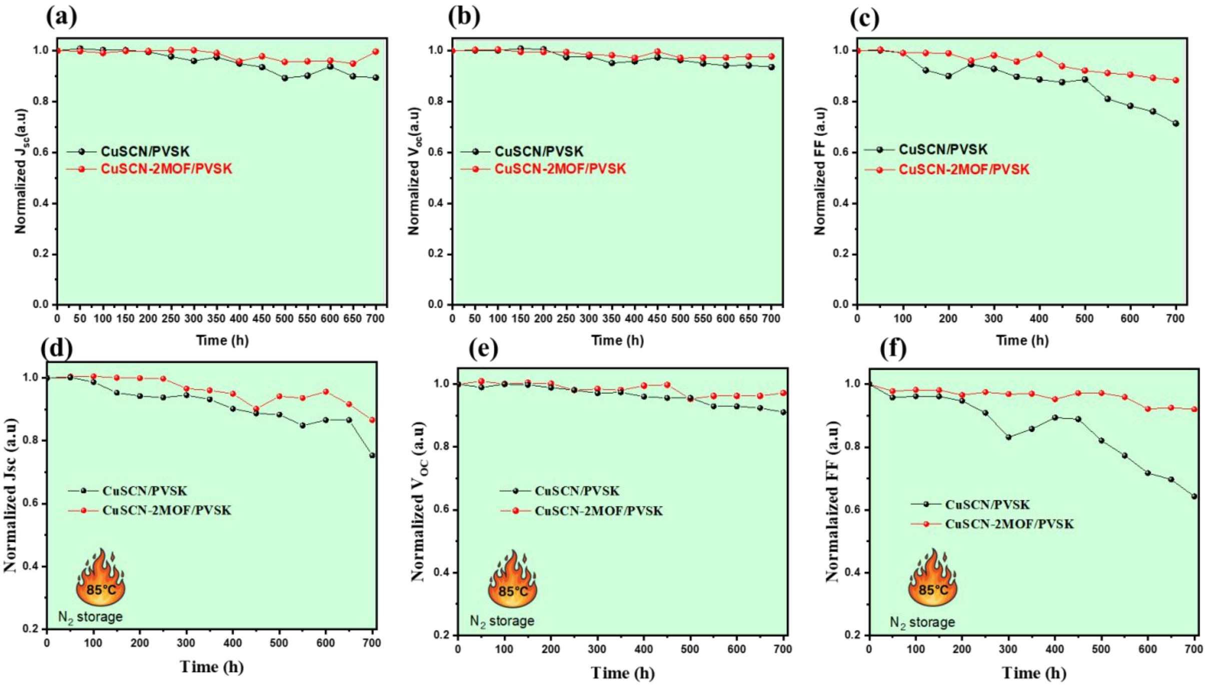

Figure S13. Evolution of J_{SC}, V_{OC}, and FF of CuSCN/PVSK and CuSCN-2MOF/PVSK based PSCs during ambient storage (a–c) and thermal aging at 85 °C under N_2 storage (d–f).

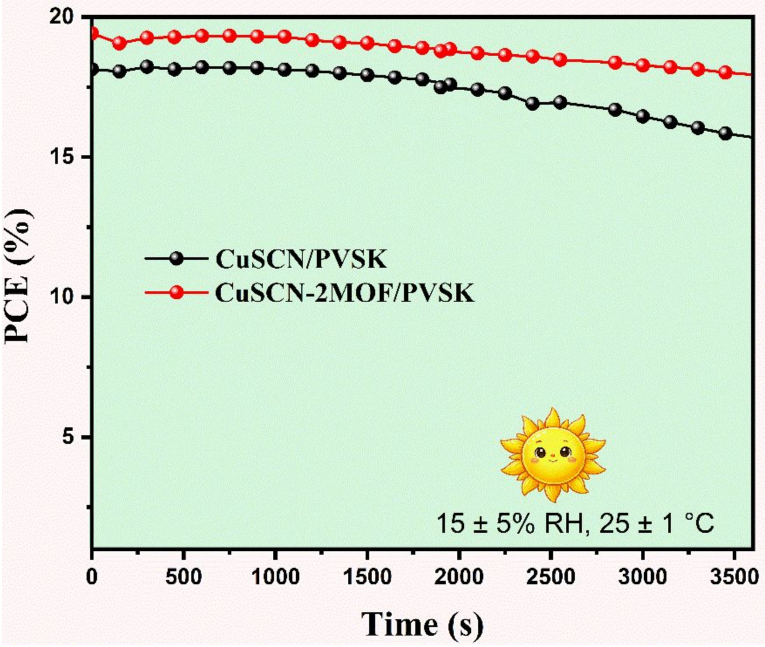

Figure S14. Continuous light-soaking test of CuSCN/PVSK and CuSCN-2MOF/PVSK-based PSCs for 1 h under ambient conditions (15 ± 5% RH, 25 ± 1 °C).

References

[1] K. Zhao, R. Munir, B. Yan, Y. Yang, T. Kim, A. Amassian, *J. Mater. Chem. A* **2015**, *3*, 20554.

[2] J. W. Jung, C.-C. Chueh, A. K.-Y. Jen, *Adv. Energy Mater.* **2015**, *5*, 1500486.

[3] S. Ye, W. Sun, Y. Li, W. Yan, H. Peng, Z. Bian, Z. Liu, C. Huang, *Nano Lett.* **2015**, *15*, 3723.

[4] N. Wijeyasinghe, A. Regoutz, F. Eisner, T. Du, L. Tsetseris, Y.-H. Lin, H. Faber, P. Pattanasattayavong, J. Li, F. Yan, M. A. McLachlan, D. J. Payne, M. Heeney, T. D. Anthopoulos, *Adv. Funct. Mater.* **2017**, *27*, 1701818.

[5] H. Wang, Z. Yu, J. Lai, X. Song, X. Yang, A. Hagfeldt, L. Sun, *J. Mater. Chem. A* **2018**, *6*, 21435.

[6] I. S. Jin, J. H. Lee, Y. W. Noh, S. H. Park, J. W. Jung, *Inorg. Chem. Front.* **2019**, *6*, 2158.

[7] M. Lyu, J. Chen, N.-G. Park, *J. Solid State Chem.* **2019**, *269*, 367.

[8] K. Ramachandran, C. Jeganathan, K. Subbian, *Nanotechnology* **2021**, *32*, 325402.

[9] P. Xu, J. Liu, J. Huang, F. Yu, R. Zhang, C.-H. Li, Y.-X. Zheng, J.-L. Zuo, *Sol. RRL* **2021**, *5*, 2000777.

[10] J. Wei, X. Guo, H. Yang, A. Liu, W. Zhang, C. Lu, X. Li, J. Fang, *J. Alloys Compd.* **2022**, *925*, 166760.

[11] J.-W. Liang, Y. Firdaus, R. Azmi, H. Faber, D. Kaltsas, C. H. Kang, M. I. Nugraha, E. Yengel, T. K. Ng, S. De Wolf, L. Tsetseris, B. S. Ooi, T. D. Anthopoulos, *ACS Energy Lett.* **2022**, *7*, 3139.
